# Microbially induced calcite precipitation by a novel alkaliphilic *Bacillus albus* strain for sustainable self-healing bio-mortar with enhanced mechanical performance and durability

**DOI:** 10.1038/s41598-026-57485-3

**Published:** 2026-06-23

**Authors:** Sahar. M. Ibrahim, Dalia Said, Mohamed Heikal, Mohamed O. Abdel-Monem, Ghada E. Dawwam

**Affiliations:** 1 Chemistry Department, Faculty of Science, Benha University, Benha, Egypt; 2 Botany and Microbiology Department, Faculty of Science, Benha University, Benha, Egypt

**Keywords:** Microbially induced calcite precipitation (MICP), *Bacillus albus*, Self-healing concrete, Bio-mortar, Compressive strength, Durability, Thermal resistance, Biotechnology, Engineering, Environmental sciences, Materials science, Microbiology

## Abstract

Micro-crack formation is a major factor limiting the durability of concrete structures. This study investigates a sustainable self-healing approach based on microbially induced calcium carbonate precipitation (MICP). Fifty bacterial isolates were obtained from alkaline soils in Wadi El-Natrun, Egypt, and screened for their ability to precipitate calcium carbonate. Isolate code W39 exhibited the highest activity, producing 0.453 g/100 mL of CaCO₃. Molecular identification by 16 S rRNA sequencing confirmed the strain as *Bacillus albus* (Accession number: PQ288981). Optimal precipitation occurred at pH 8, with 25 mM CaCl₂ and 20 g/L urea, after seven days of incubation at 30 °C. Instrumental analyses, including scanning electron microscopy (SEM) coupled with an energy-dispersive X-ray (EDX) analyzer, high-resolution transmission electron microscopy (TEM), Fourier-transform infrared (FT-IR) spectroscopy, X-ray diffraction (XRD), and the N₂ desorption/adsorption isotherm (BET) method, verified that the biogenic CaCO₃ consisted of nanoscale, high-purity calcite. The self-healing potential was evaluated by embedding viable *Bacillus albus* cells in cement mortar at optical densities (OD₆₀₀) of 0.5, 1.0, and 1.5, and curing in urea-enriched media with varying CaCl₂ concentrations (25–100 mM). Bacterial incorporation significantly enhanced mechanical performance over a 90-day curing period. The optimal dosage depended on Ca²⁺ availability: OD₆₀₀ 1.5 provided the greatest improvement at 25 mM CaCl₂, whereas OD₆₀₀ 0.5 was more effective at 50–100 mM. Microstructural analyses, comprising x-ray diffraction (XRD), differential thermal/thermogravimetric analysis (DTG/TGA), and scanning electron microscopy (SEM), confirmed the formation of additional C–S–H and calcite, resulting in reduced porosity and a denser matrix. The bio-mortar also demonstrated increased durability, including resistance to exposure to MgSO₄ and MgCl₂, as well as thermal stability up to 1000 °C. These findings demonstrate that the indigenous *Bacillus albus* strain is a promising agent for durable, self-healing bio-concrete. Overall, these results show that microbial incorporation improves the pore system, encourages the formation of more hydrates and CaCO_3_, and creates a more resilient mortar that can withstand extreme thermal exposure.

**Supplementary Information:**

The online version contains supplementary material available at 10.1038/s41598-026-57485-3.

## Introduction

Concrete is the most widely used construction material globally, second only to water in total consumption, yet its reliance on cement production contributes significantly to environmental burdens, accounting for approximately 8–10% of global anthropogenic CO₂ emissions^1^. Despite its widespread use, concrete’s inherently low tensile strength makes it highly susceptible to cracking, compromising structural performance and necessitating frequent maintenance. Conventional repair methods, such as epoxy injection, are often energy-intensive, environmentally unfriendly, and prone to incompatibility with the surrounding matrix, which can induce secondary cracking and result in high long-term failure rates^2,3^. These limitations highlight the urgent need for autonomous, durable, and environmentally sustainable repair strategies.

In response to the growing demand for environmentally responsible construction materials, self-healing technologies have attracted significant interest, with microbially induced calcium carbonate precipitation (MICP) emerging as a promising strategy. This bio-mediated process utilizes microorganisms as renewable agents capable of autonomously sealing cracks, providing a sustainable and efficient alternative to conventional repair approaches^2^.

MICP offers a viable solution to this challenge by utilizing ureolytic bacteria capable of hydrolyzing urea, thereby increasing local pH and inducing the precipitation of calcite (CaCO₃) within cracks. This biogenic process effectively seals micro-cracks, enhances mechanical performance, and reduces permeability^4,5^. During precipitation, the bacterial cell surface functions as a nucleation site, attracting Ca^2+^ and facilitating the formation of CaCO₃, a chemically compatible and durable mineral that integrates efficiently with the cementitious matrix^6^. Although model strains such as *Bacillus sphaericus* and *Sporosarcina pasteurii* have been extensively investigated^7^. Ongoing research continues to explore more resilient, alkaliphilic, and high-yielding isolates from extreme environments to further advance and optimize MICP-based self-healing technologies.

A major challenge in the application of MICP-based self-healing systems is ensuring the survival and metabolic activity of bacterial cells within the highly alkaline environment of concrete and the harsh conditions associated with cement hydration and mixing. This highlights the importance of employing spore-forming, alkaliphilic strains isolated from naturally high-pH ecosystems, which are inherently more capable of withstanding such conditions. In cementitious matrices, these bacteria can persist in a dormant state during the early stages of hydration and mechanical processing, and are subsequently activated upon crack formation and water ingress, enabling effective MICP-based calcium carbonate precipitation under service conditions^8^. Moreover, the overall efficiency of MICP depends not only on the intrinsic properties of the bacterial strain but also on the interplay between cell concentration and the availability of chemical precursors, particularly Ca^2+^ ions. A comprehensive understanding of this interaction is essential for maximizing healing performance^9^.

Bacterial incorporation is pivotal for survival and performance in cementitious systems, achieved mainly through direct addition or encapsulation. In the direct method, cells or spores are mixed into concrete or applied to cracks, with outcomes depending on viability, concentration, and resistance to alkaline and mechanical stresses. Higher concentrations enhance urease activity and CaCO₃ precipitation, though survival remains limited, making spore-forming *Bacillus* species preferable. Encapsulation, by contrast, protects bacteria within carriers during storage, mixing, and hydration. Upon crack formation and water ingress, capsules release bacteria to precipitate CaCO₃, thereby improving protection, healing efficiency, and long-term durability^2,6^.

The long-term durability of concrete is strongly affected by exposure to aggressive agents such as SO_4_^2−^, and Cl^−^, which infiltrate cracks and pore networks, degrade the cementitious matrix, and accelerate corrosion of steel reinforcement^10^. Harsh service environments, including fire, freeze–thaw cycling, and corrosive conditions associated with sewage or seawater, have prompted extensive research on durability-related properties such as high-temperature resistance, freeze–thaw stability, impermeability, and chemical attack resistance^11^. Incorporating microorganisms through MICP significantly enhances durability by sealing micro-cracks and refining the pore structure via CaCO₃ deposition, thereby decreasing permeability and strengthening resistance to chemical degradation, particularly against SO_4_^2−^, and Cl^−^ intrusion^12^.

Sulphate attack is one of the most widespread and damaging forms of concrete deterioration, resulting in reduced long-term durability, internal expansion, and strength degradation^13^. This phenomenon occurs when external SO_4_^2−^ ions penetrate the concrete matrix and react with key hydration products, particularly calcium silicate hydrate (C–S–H), portlandite (CH), and tricalcium aluminate phase (C₃A). These reactions lead to the formation of secondary ettringite, whose crystallization within the pore structure generates expansive stresses, promotes microcracking, and accelerates structural deterioration^14,15^. Chloride attack produces non-expansive compounds such as Friedel’s salt. Upon ingress, Cl^−^ ions partly immobilize through binding with hydration products. However, MgCl₂, commonly applied as a deicing salt in cold climates, introduces both Mg²⁺ and Cl⁻ ions into the pore network^16^. These ions interact with cementitious phases through diffusion or absorption, progressively undermining the mechanical and durability performance of concrete^17^.

Fire exposure constitutes a major hazard for concrete structures, as elevated temperatures trigger a series of physicochemical transformations that severely compromise mechanical performance. Thermal degradation begins with the loss of interlayer and chemically bound water from C–S–H, followed by the decomposition of CH and progressive breakdown of C–S–H phases^18^. These alterations induce extensive microstructural damage, leading to substantial reductions in compressive strength, often by about 50% at 600 °C and nearly complete loss at temperatures exceeding 800 °C^19^. In Portland cement systems, Ca(OH)₂ dehydrates to CaO between 450 and 550 °C; upon cooling, the rehydration of CaO in the presence of atmospheric moisture causes approximately 40% volumetric expansion, generating cracking and further destabilizing the matrix^20^. Additional thermal transformations influence durability, including the dehydration of ettringite at 130–170 °C, the α–β phase transition of quartz aggregates at 573 °C^21^, and the onset of limestone decarbonation at roughly 700 °C^22^. Partial decomposition of C–S–H at moderate temperatures contributes to the formation of CaCO₃ and β-C₂S, while complete dehydration between 100 and 450 °C ultimately yields C₂S and C₃S at temperatures above 600–800 °C^23^.

Incorporation of bacteria through MICP markedly enhances concrete durability by sealing microcracks and refining pore structures through CaCO₃ deposits, thereby decreasing permeability and improving resistance against SO_4_^2−^, and Cl^−^ intrusion. Although numerous studies have investigated microbially induced calcium carbonate precipitation (MICP) using conventional ureolytic bacteria such as *Sporosarcina pasteurii* and *Bacillus sphaericus*, these microorganisms often exhibit limited survivability under the highly alkaline and chemically aggressive conditions of cementitious environments. Comparatively limited attention has been directed toward indigenous alkaliphilic bacteria isolated from extreme natural habitats and their long-term performance within concrete systems. Moreover, most previous investigations have primarily focused on crack-healing efficiency or short-term mechanical enhancement, whereas comprehensive assessments involving bacterial optimization, hydration evolution, microstructural densification, resistance to sulfate and chloride attack, and thermal stability remain insufficiently explored. In addition, the combined influence of bacterial concentration and calcium ion availability on the multifunctional durability performance of bio-concrete has not yet been systematically evaluated. Accordingly, the present study addresses these limitations through the isolation and application of a novel alkaliphilic spore-forming bacterial strain, *Bacillus albus*, isolated from the extreme alkaline environment of Wadi El-Natrun. The proposed approach integrates microstructural, mechanical, chemical, and thermal durability analyses to comprehensively evaluate the performance of the developed bio-cementitious system. Unlike earlier studies that mainly relied on model bacterial strains with limited tolerance to harsh concrete conditions, the current work emphasizes the utilization of naturally adapted alkaliphilic microorganisms capable of surviving and functioning effectively within cementitious matrices. Furthermore, while most reported studies have concentrated on short-term mechanical improvements, relatively few investigations have addressed long-term durability under sulfate/chloride exposure and elevated-temperature conditions. Therefore, this work highlights the necessity of isolating and characterizing novel alkaliphilic, spore-forming bacteria from extreme environments to enhance the long-term multifunctional durability and sustainability of bio-based cementitious materials.

Building on this gap, the present study hypothesizes that a novel alkaliphilic bacterium isolated from the extreme environment of Wadi El-Natrun, Egypt, will exhibit high calcifying efficiency, promote the formation of a denser microstructure, and substantially improve mortar strength, resistance to chemical attack, and stability under elevated temperatures. The novelty of this work is threefold. First, it introduces the isolation and application of a new strain of *Bacillus albus* from Wadi El-Natrun, selected for its exceptional survivability and activity within the highly alkaline concrete matrix. Second, it provides an in-depth examination of the synergistic interaction between bacterial concentration and calcium ion availability, identifying the optimal conditions required to maximize healing efficiency. Third, it presents a comprehensive performance evaluation demonstrating the bio-mortar’s superior durability against SO_4_^2−^, and Cl^−^ exposure, alongside enhanced thermal resistance up to 1000 °C. Accordingly, this study aims to isolate and characterize robust calcifying bacteria, establish the optimal parameters for CaCO₃ precipitation, and assess the resulting bio-mortar’s physico-mechanical properties and long-term durability.

## Materials and methods

### Sampling location and collection

In November 2023, five soil samples were collected from distinct sites surrounding the saline and highly alkaline lakes of Wadi El-Natrun, Egypt, a sandy depression situated in the western Nile Delta (30°17′–30°38′ N, 30°02′–30°30′ E) at approximately 22 m below sea level. From each sampling point, approximately 500 g of soil was obtained at a depth of 10–15 cm after the removal of surface debris and coarse materials. The samples were transferred into sterile, airtight containers, transported under controlled conditions, and subsequently stored at 4 °C to maintain bacterial viability before isolation and characterization.

### Isolation and cultivation of calcifying bacteria

Ten grams of each soil sample were inoculated into 100mL of B4 medium (per liter: 4 g yeast extract, 5 g dextrose, 2.5 g calcium acetate, and 1.5–2% agar; pH 8.2)^24^. To avoid thermal decomposition, calcium acetate was sterilized separately by syringe filtration and added aseptically after autoclaving the remaining components. The inoculated cultures were incubated at 30 °C with shaking at 150 rpm for 7 days to promote bacterial enrichment. Serial dilutions (10⁻¹ to 10⁻⁶) were subsequently prepared, and the final three dilutions were spread-plated onto B4 agar to obtain isolated colonies. Colonies exhibiting white crystalline deposits, presumptive indicators of calcium carbonate precipitation, were repeatedly sub-cultured to achieve pure isolates. For long-term preservation, purified strains were stored in 20% glycerol at − 80 °C.

### Screening of purified isolates for high-yield CaCO₃ production

The purified isolates were cultured in 100mL of B4 broth (pH 7.0) in triplicate and incubated at 30 °C with shaking at 150 rpm for 7days. Non-inoculated flasks served as negative controls. Following incubation, the precipitated CaCO₃ was collected, washed, dried, and quantified according to the Standard Precipitation Protocol.

Standard Precipitation Protocol: After incubation, cultures were centrifuged at 8000 rpm for 10 min at 4 °C. The resulting pellets were washed twice with sterile distilled water and dried at 105 °C to a constant weight^24^. The CaCO₃ yield was then calculated using the following equation.

1 $$W_{{precipitates}} = W_{{Total}} - W_{{Paper~~}}$$

where $\:{W}_{\mathrm{precipitates}}\:$is, the weight of CaCO₃ collected from inoculated flasks, $\:{W}_{\mathrm{Total}}\:$is the measured weight of filter paper containing precipitates, and $\:{W}_{\mathrm{Paper}}\:$presents the weight of empty filter paper. Strains demonstrating the highest CaCO₃ yield were selected for subsequent characterization.

### Preliminary identification

#### Morphological characterization and biochemical analysis

The selected bacterial isolate was subjected to microscopic examination using Gram staining, following the procedure described by Wu et al.^25^. Sporulation ability was assessed by incubating the culture in nutrient medium at 30 °C with shaking at 150 rpm for 24–48 h, followed by heat treatment at 80 °C for 1 h to eliminate vegetative cells. Subsequent re-culturing on nutrient agar confirmed the presence of heat-resistant spores^26^. Transmission electron microscopy (TEM) was performed to examine the morphological features of the isolated bacterial strain before application in the cementitious system. Urease activity was evaluated by inoculating the isolate into 15mL of lysogeny broth (LB) supplemented with 0.012 g/L phenol red and 20 g/L urea, adjusted to pH 6.8. Following incubation at 37 °C for 24–48 h, the appearance of a pink coloration indicated positive urease activity^27^. Catalase activity was determined according to the protocol outlined by Loewen et al.^28^.

Molecular Identification of Selected Bacterial Isolate:

Genomic DNA of isolate coded W39 was extracted using the Gspin™ Total Extraction Kit in accordance with the manufacturer’s instructions. The 16 S rRNA gene was amplified using universal primers 27 F (5′-AGA GTT TGA TCM TGG CTC AG-3′) and 1492R (5′-TAC GGY TAC CTT GTT ACG ACT T-3′) on a Biometra thermocycler, following the protocol of Dawwam et al.^29^. The PCR program consisted of an initial denaturation at 95 °C for 5 min, followed by 30cycles of denaturation at 95 °C for 30s, annealing at 55 °C for 2 min, and extension at 72 °C for 1 min, with a final extension at 72 °C for 10 min. Amplified products were purified using the Montage PCR Clean-up Kit (Millipore). Sequencing was conducted using the Big Dye Terminator Cycle Sequencing Kit v3.1 and analyzed on an Applied Biosystems 3730XL platform at Macrogen Inc. (South Korea). The resulting sequence was compared to the GenBank database using BLAST, and identification of the isolate was based on the highest sequence similarity.

### Optimization of CaCO₃ precipitation

The optimal conditions for CaCO₃ precipitation by the selected isolate (W39) were determined by varying one parameter at a time using a modified Urea–CaCl₂ broth. Following a 7-day incubation period, CaCO₃ production was quantified using the Standard Precipitation Protocol. The parameters evaluated included:

- pH: The initial medium pH was adjusted to values between 6 and 11.
- CaCl₂ Concentration: CaCl₂ concentrations ranging from 0.01 to 1 mol/L were tested.
- Incubation Period: Cultures were incubated for 1 to 7 days to assess temporal effects on CaCO₃ precipitation.
- Temperature: Incubation temperatures of 10, 20, 30, and 40 °C were evaluated.

###  Characterization of precipitated CaCO_3_

The crystalline phases and mineral composition of the precipitated CaCO₃ were characterized using X-ray powder diffraction (XRD). Analyses were performed on finely ground samples with a Philips 1830/40 diffractometer equipped with Cu–Kα radiation (40 kV, 30 mA) and a Ni filter, operating at a scanning rate of 0.005°2θ s⁻¹. A time constant of 2s was applied, and diffraction patterns were collected over the 2θ range of 10°–60°. Particle size, morphology, and microstructural features were examined using field-emission scanning electron microscopy (FE-SEM, Jeol JMS-700) operating at 10 kV, coupled with an energy-dispersive X-ray (EDX) analyzer for elemental composition. Nanoparticle characteristics and crystallinity were further investigated using high-resolution transmission electron microscopy (HR-TEM, Jeol JEM-2100, Japan).

Fourier-transform infrared (FT-IR) spectroscopy was conducted using a Genesis-II FT-IR spectrometer on KBr-disc samples. Spectra were recorded over the wavenumber range of 400–4000 cm⁻¹ at the Chemistry Department, Faculty of Science, Benha University. The specific surface area of the precipitated CaCO₃ was determined from N₂ adsorption–desorption isotherms using the Brunauer–Emmett–Teller (BET) method with a NOVA touch LX2 surface area and pore size analyzer (model NT2LX-2, Quantachrome, USA).

### Direct incorporation of bacterial isolate in bio-mortar

#### Applicable raw materials

Ordinary Portland Cement (OPC, Type I, 42.5 N), commercially known as Al Momtaz and manufactured by Lafarge Cement Company, Egypt, was used throughout this study. The oxide composition of the OPC was determined using X-ray fluorescence spectroscopy (XRF, PW-1400), and the results are presented in Table 1.

**Fig. 1:**
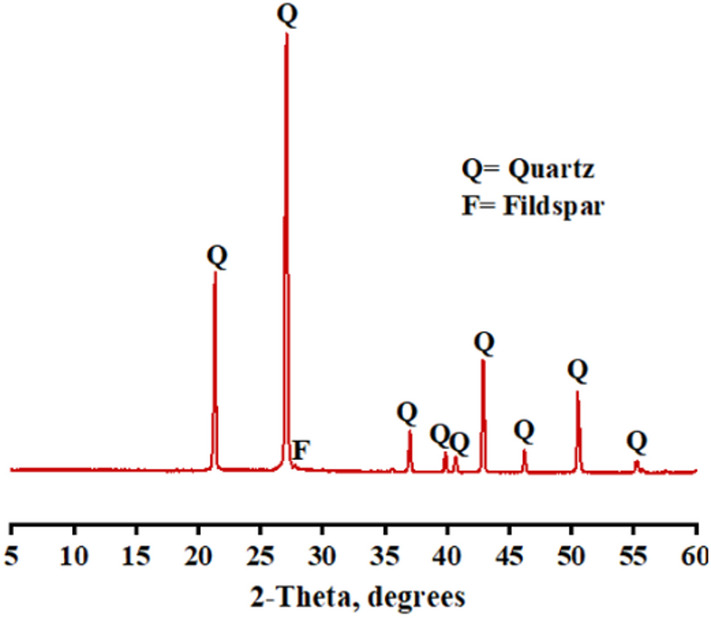
XRD patterns of standard sand.

Standard sand of French origin, supplied by Société Nouvelle du Littoral (SNL), France, was employed in accordance with CEN EN 196-1 and ISO 679:2009 specifications for mortar preparation. The mineralogical composition of the standard sand is shown in Fig. 1. As illustrated, the sand consists predominantly of quartz, with minor amounts of feldspar.

**Table 1:** Chemical oxide composition of OPC (wt., %).

Oxides	CaO	Al_2_O_3_	SiO_2_	Fe_2_O_3_	Na_2_O	K_2_O	SO_3_	Cl^−^	MgO	L.O. I
Mass,%	64.11	4.65	19.4	3.82	0.31	0.27	3.16	0.0	1.85	2.43

L.O.I = loss of ignition.

#### Microbial enhanced-cement mortar preparation

Mortar specimens were prepared to assess the influence of bacterial incorporation on the physicochemical characteristics of the mortar. A constant W/C = 0.4 was used for all mixtures, with OPC/standard sand ratio = 1:2 by weight. The dry components were homogenized thoroughly, after which the actively grown bacterial culture was added at OD₆₀₀: 0.5, 1.0, and 1.5. Control specimens were prepared using distilled water in place of the bacterial suspension. The detailed mix proportions are presented in Table 2.

Freshly prepared mixtures were cast into cylindrical molds (1-inch diameter × 2 inches height). The molds were vibrated for several minutes to eliminate entrapped air and ensure adequate compaction. Following casting, the specimens were maintained under controlled conditions at ambient temperature and RH = 98 ± 1% for the first 24 h. After the initial curing period, the specimens were demolded and immersed in curing solutions containing 20 g/L urea and CaCl₂ at concentrations of 25, 50, or 100mM. The solutions were periodically refreshed, and the specimens were stored at room temperature (≈ 28 °C) until the designated curing intervals of 1, 3, 7, 28, and 90 days.

**Table 2:** The designations, mix compositions, and water/cement ratio.

Mix abb.	OPC (g)	Standard sand (g)	Bacterial Solution Conc. OD_600_)	CaCl_2_ Conc. mM)	W/C ratio
B0-C25	100	200	0.00	25	0.4
B0-C50	100	200	0.00	50	0.4
B0-C100	100	200	0.00	100	0.4
B1-C25	100	200	0.50	25	0.4
B1-C50	100	200	0.50	50	0.4
B1-C100	100	200	0.50	100	0.4
B2-C25	100	200	1.00	25	0.4
B2-C50	100	200	1.00	50	0.4
B2-C100	100	200	1.00	100	0.4
B3-C25	100	200	1.50	25	0.4
B3-C50	100	200	1.50	50	0.4
B3-C100	100	200	1.50	100	0.4

#### Methods of investigation

A set of three cylindrical specimens was used to determine the compressive strength (CS) of the cement mortars after curing periods of 1, 3, 7, 28, and 90 days, in accordance with Testing and Cement^30^. Compressive strength measurements were performed following ASTM C109M using a Seidner Riedlinger testing machine (West Germany), with a maximum capacity of 600kN and a loading rate of approximately 0.35–0.70 MPa/s. After CS testing, the fractured pieces from each cylinder were collected for additional analyses. Chemically combined water content (Wn), bulk density (BD), and total porosity (TP) were determined according to the procedures described by Said et al.^31^.

To evaluate durability, mortar specimens were prepared using bacterial suspensions at OD₆₀₀ = 0.5. After casting and demolding, the samples were immersed in curing solutions containing 20 g/L urea and CaCl₂ concentrations of 25, 50, or 100mM for 28 days (0 days). Specimens were then dried at 105 °C for 24 h before exposure to aggressive chemical environments. The dried samples were immersed in 5%MgSO₄ or 5%MgCl₂ solutions for 0 days, 7, 14, 21, 28, and 60 days. To maintain consistent chemical concentrations, the sulphate and chloride solutions (5% MgSO₄ or 5% MgCl₂) were refreshed weekly. Total sulphate and total chloride contents were determined according to the procedures described by Sato et al.^32^ and Heikal et al.^33^.

After 28 days of curing, fire resistance testing was conducted. Three cylinders from each mixture were placed in a furnace, heated at a controlled rate of 2 °C/min, and maintained for 2 h at each target temperature (200, 400, 600, 800, and 1000 °C). Following heating, the furnace was switched off to allow gradual cooling. The cylinders were subsequently weighed to determine weight loss using Eq. (2):

2 $$Firing{\text{ }}weight{\text{ }}loss\% = ~\frac{{weight~before~igination - weight~after~igination}}{{ignited~weight}} \times 100$$

The compressive strength of the heat-treated sample was determined at each temperature, such as 200, 400, 600, 800, and 1000 °C.

**Scheme 1:**
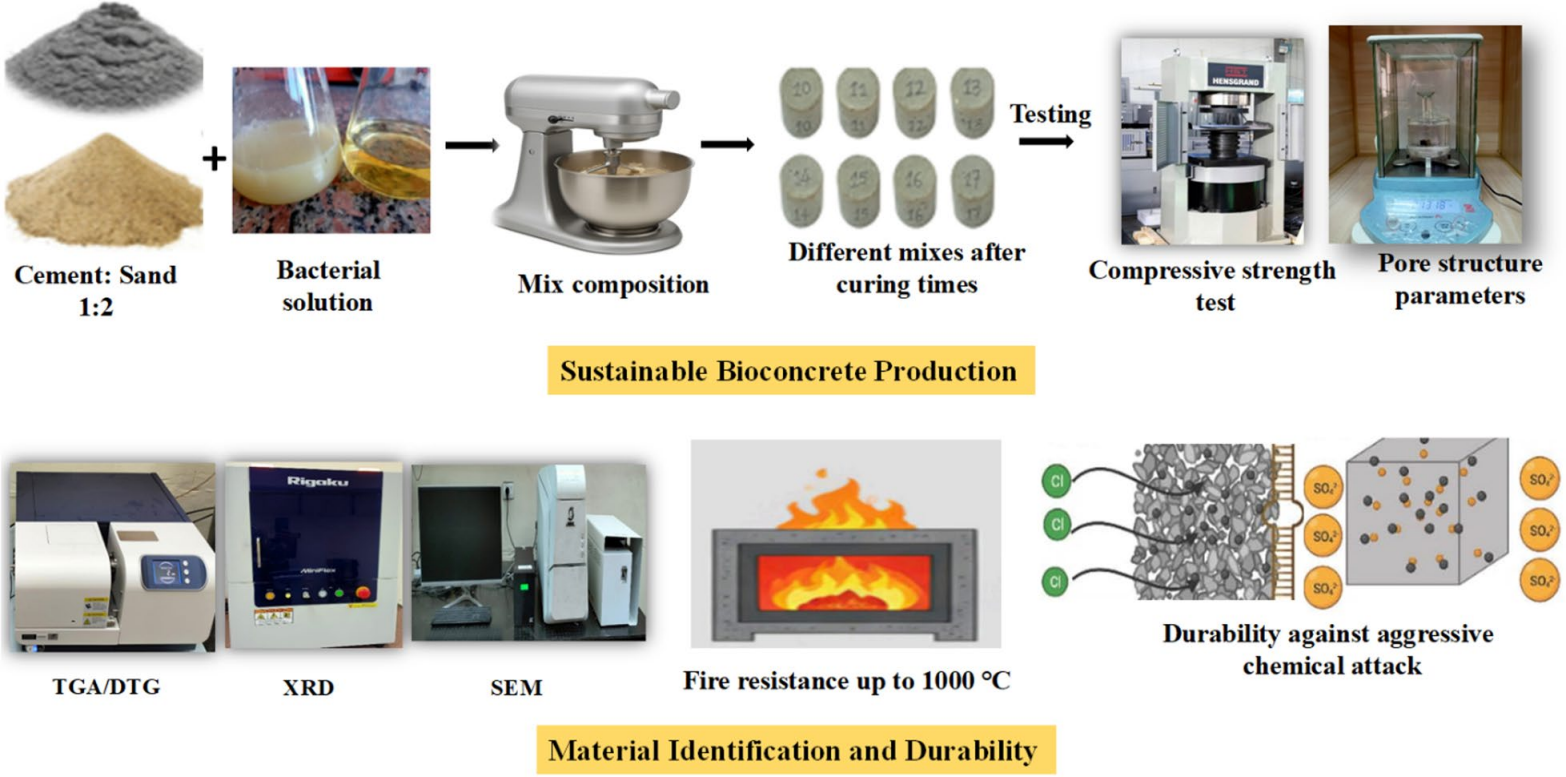
Integrated workflow for sustainable bio-concrete production and durability assessment.

Phase Identification

The phase composition of the hydration products was analyzed using differential thermogravimetric analysis (DTG/TGA) and X-ray diffraction (XRD) to identify both reacted and unreacted phases in selected samples. Thermogravimetric and differential thermal analyses (TGA/DTA) were performed using a simultaneous thermal analyzer (NEXTA STA 200, Japan). Thermal tests were conducted from room temperature (23 ± 2 °C) up to 1000 °C at a heating rate of 10 °C/min under a nitrogen atmosphere, using ceramic crucibles.

XRD measurements were carried out using a Philips 1830/40 diffractometer equipped with Cu–Kα radiation (40 kV, 30 mA) and a nickel filter. Diffraction data were collected at a scanning speed of 0.005° 2θ/s with a time constant of 2s, over a 2θ range of 10°–60°. The morphology of the hydration products and microstructural features within the cured mortar matrix was further examined using SEM. A schematic overview of the experimental workflow is presented in Scheme 1.

## Results and discussion

### Isolation and screening for CaCO_3_-producing bacteria

**Fig. 2:**
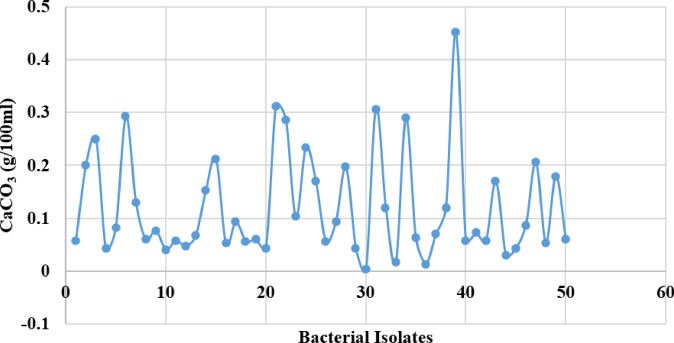
Calcium carbonate production (g/100 ml) by bacterial isolates.

The data presented in Fig. 2 and Supplementary Table 3 demonstrate substantial variability in CaCO₃ precipitation among the bacterial isolates cultured in B4 broth. Isolates W39, W21, and W31 produced the highest CaCO₃ yields, 0.453, 0.313, and 0.307 g/100 mL, respectively, and were also characterized by elevated final pH values (≥ 9.05) and strong growth performance. These observations are consistent with previous reports indicating that higher pH environments increase carbonate ion availability, thereby promoting CaCO₃ precipitation^34^.

A positive correlation was observed between bacterial growth (OD₆₀₀ absorbance) and CaCO₃ yield, suggesting that metabolically active cells contribute more effectively to biomineralization. Ureolytic bacteria elevate medium pH through urea hydrolysis, creating supersaturated conditions that accelerate calcite nucleation and growth^35^. Isolates displaying moderate to high growth levels ( + + to +++) consistently produced greater amounts of CaCO₃, reinforcing the central role of urease activity in the MICP process.

Notably, isolates recovered from the third soil sample (e.g., W21, W22, W24, W25) exhibited superior performance, possibly reflecting adaptation to the extreme salinity and alkalinity characteristic of Wadi El-Natrun. Such habitats tend to favor the proliferation of alkaliphilic, urease-positive strains capable of efficient bio-cementation^36^. In contrast, isolates from the fourth soil sample (e.g., W29, W30, W33, W36) displayed lower final pH values (≤ 6.65) and negligible CaCO₃ precipitation (< 0.05 g/100 mL), likely indicating weak ureolytic activity or suboptimal metabolic compatibility with the medium. Thus, these findings highlight the critical influence of strain selection and environmental provenance on MICP efficiency. The superior performance of isolates W39, W21, and W31 underscores their potential suitability for incorporation into bio-concrete formulations designed to enhance durability through autonomous self-healing mechanisms.

###  Morphological and biochemical characterization of bacterial isolates

Isolate W39 exhibited morphological and biochemical characteristics typical of calcite-precipitating, ureolytic bacteria. Gram staining identified the organism as a Gram-positive, rod-shaped bacterium arranged in chains, features commonly associated with *Bacillus* species known to participate in MICP^37^. Catalase activity was confirmed through rapid bubble formation upon exposure to hydrogen peroxide, indicating enzymatic decomposition into water and oxygen. Such activity reflects adaptation to oxidative stress and compatibility with alkaline, calcium-rich environments characteristic of MICP systems^38^.

The isolate also tested positive for urease activity, as evidenced by the color change from yellow to pink in LB medium. Urease catalyzes the hydrolysis of urea into ammonia and carbon dioxide, increasing the pH and generating carbonate ions required for CaCO₃ precipitation^27^.

Further analysis confirmed that W39 is spore-forming. Under high-pH conditions, vegetative cells can transition into dormant spores and later revert to active growth when favorable conditions return^39^. This behavior is further supported by TEM images, which reveal morphological features consistent with a spore-forming *Bacillus* strain, supporting its identification and structural integrity (Supplementary Fig. 1). These findings corroborate the well-established role of urease-producing bacteria in biomineralization and highlight their relevance for bio-concrete enhancement^40^.

###  Molecular identification of selected bacterial isolates

**Fig. 3:**
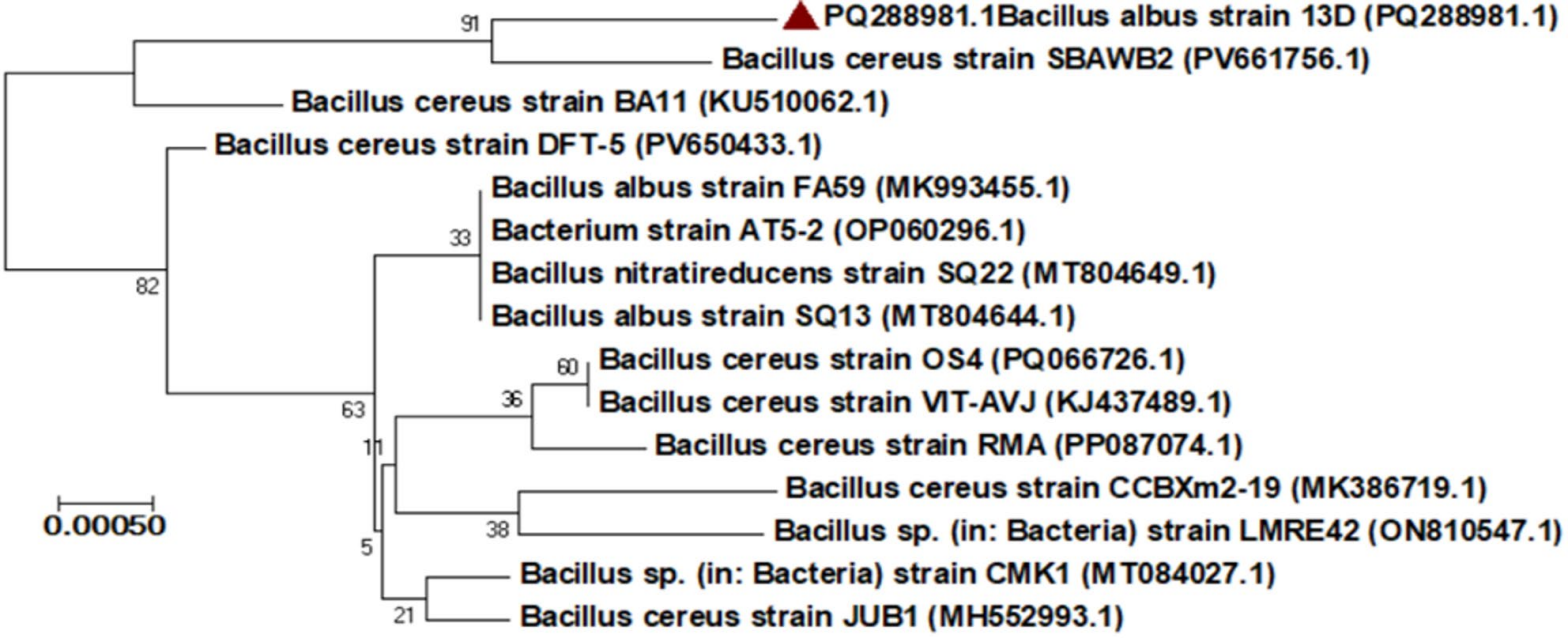
Phylogenetic tree of isolate W39 based on 16 S rRNA gene sequences, constructed using the maximum composite likelihood method. Evolutionary analyses were performed with MEGA7.

Figure 3 illustrates the phylogenetic analysis of isolate W39, highlighting its taxonomic affiliation and genetic relatedness to closely associated *Bacillus strains*. Molecular identification based on 16 S rRNA gene sequencing confirmed that W39 belongs to *Bacillus albus*, a species recognized for its biomineralization capabilities^41^. The sequence has been deposited in the GenBank database under accession number PQ288981, thereby supporting its distinctiveness and enabling future phylogenetic and comparative genomic studies. This molecular characterization further substantiates the relevance of W39 in MICP and underscores its potential application in bio-concrete technologies aimed at enhancing durability and self-healing performance.

### Optimization of different factors for high-yield CaCO_3_ precipitation

**Fig. 4:**
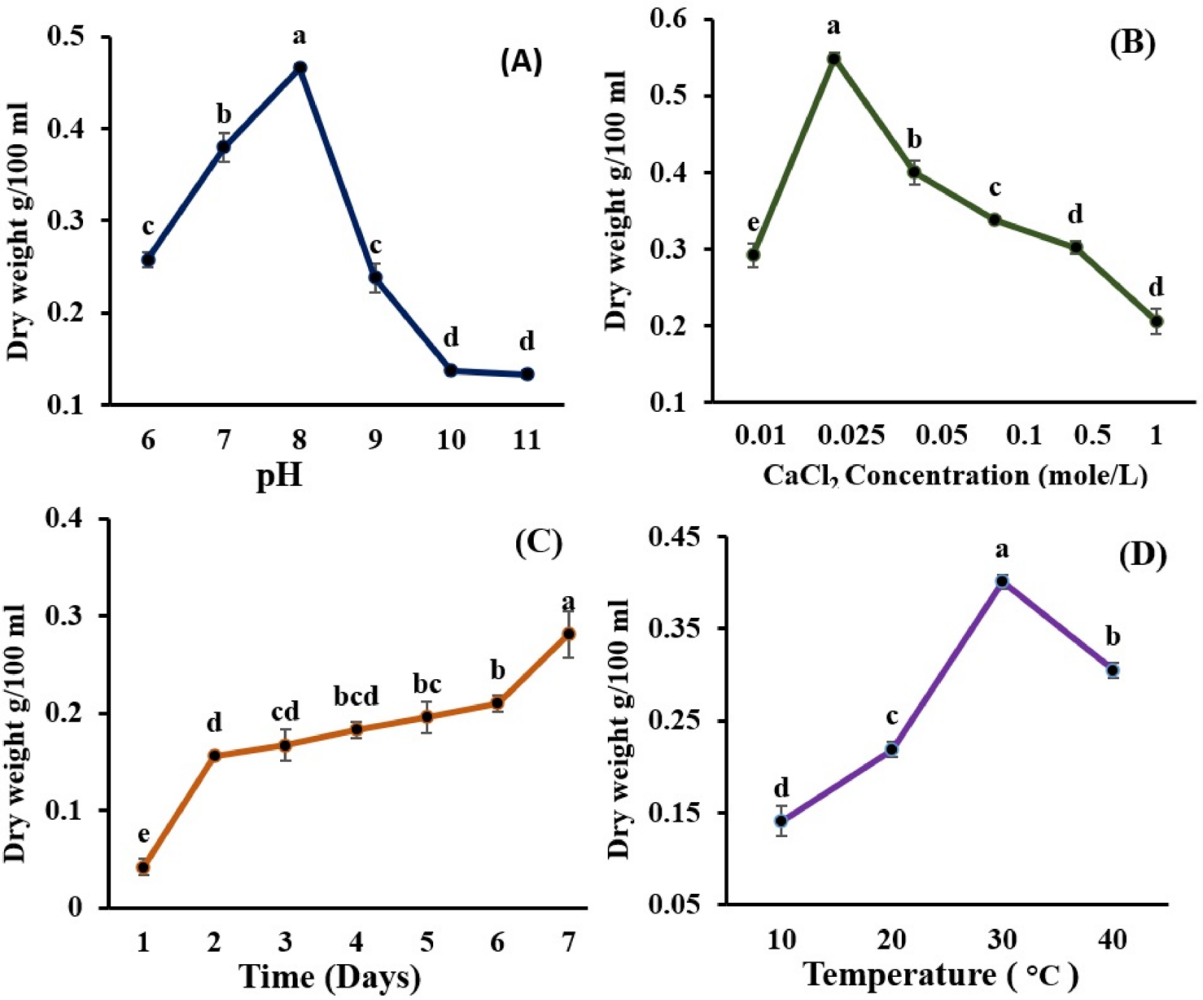
Factors influencing CaCO₃ precipitation by isolate W39: (**A**) pH; (**B**) CaCl₂ concentration; (**C**) incubation period; and (D) temperature. Different letters above the columns indicate significant differences (*p* < 0.05) according to Duncan’s multiple range test. Error bars represent the standard deviation (± SD) of triplicate measurements.

Dry weight reached its maximum at pH 8 (0.48 g/100 mL), indicating that this moderately alkaline condition is optimal for CaCO₃ precipitation (Fig. 4A). This result supports the established role of moderate pH in enhancing urease activity and increasing carbonate ion availability, both essential factors for efficient MICP^27^. At higher pH levels (≥ 10), CaCO₃ yield declined markedly, likely due to enzyme inhibition or microbial stress, consistent with reported pH-induced limitations in biomineralization^38^.

The effect of calcium ion concentration also followed a distinct trend: the highest dry weight (0.55 g/100 mL) was observed at 25 mM CaCl₂, indicating that this concentration provides optimal Ca²⁺ availability for precipitation (Fig. 4B). This aligns with findings for other ureolytic species, where moderate calcium concentrations maximize precipitate formation. At elevated concentrations, yield declined sharply; for example, 1 M CaCl₂ yielded only ~ 0.22 g/100 mL, suggesting that excessive Ca²⁺ may disrupt cellular metabolism or interfere with orderly crystal formation. Similar reductions in MICP efficiency at high calcium levels have been attributed to osmotic stress and cytotoxic effects^42^.

Dry weight increased progressively throughout the 7-day incubation period, reaching 0.38 g/100 mL on day 7 (Fig. 4C). This trend reflects sustained microbial proliferation and urease-mediated carbonate accumulation, underscoring the importance of adequate incubation time for maximizing biomineralization^27^.

Temperature exerted a pronounced influence on CaCO₃ precipitation, with optimal performance at 30 °C (0.42 g/100 mL) (Fig. 4D). Reduced yields at 10 °C and 40 °C indicate that temperatures outside this moderate range impair microbial viability and enzymatic activity, consistent with previous observations on temperature sensitivity in MICP systems^43^. Collectively, these results highlight the necessity of optimizing environmental parameters, including pH, Ca²⁺ concentration, incubation duration, and temperature, to achieve maximum MICP efficiency for bio-concrete applications.

### Characterization of precipitated CaCO_3_

The nanoscale morphology and dispersion of the precipitated CaCO₃-nanoparticles were characterized using HR-TEM (Supplementary Fig. 2) and FE-SEM (Supplementary Fig. 3). The crystalline structure of the CaCO₃ produced by isolate W39 was confirmed by XRD, as shown in Supplementary Fig. 3B. Supplementary Fig. 4 presents the FT-IR spectra of the CaCO₃ precipitates, providing additional insight into their functional groups and mineralogical composition. The N₂-adsorption–desorption isotherms and corresponding pore size distribution curves of the precipitated CaCO₃ are shown in Supplementary Fig. 5, offering detailed information on surface area and textural properties.

### Characterization methods of bio-modified mortar

#### Compressive strength

**Fig. 5:**
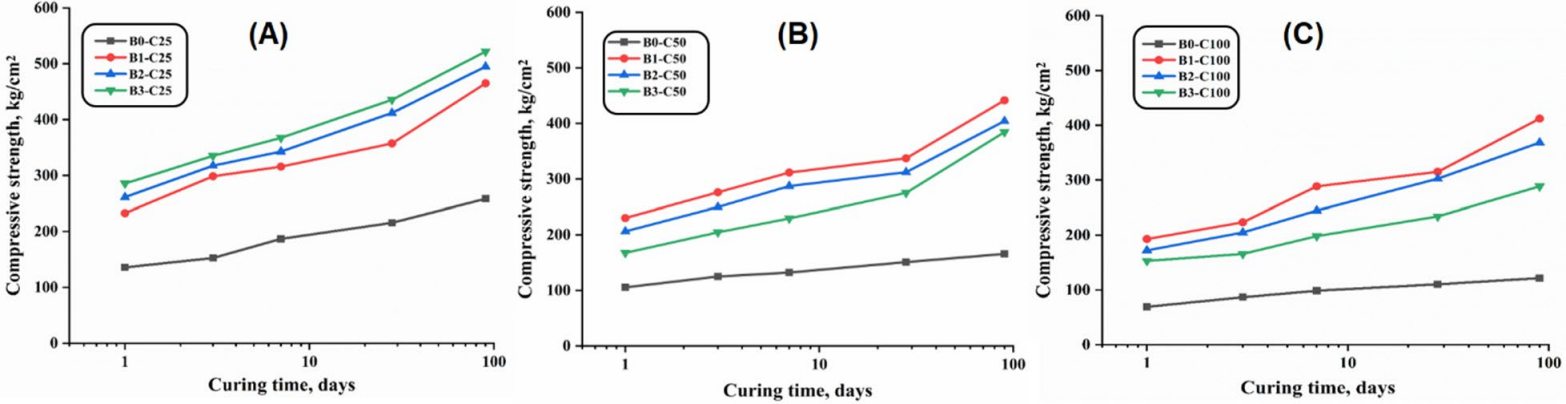
Compressive strength of bio-mortar specimens treated with (**A**) 25 mM CaCl₂; (**B**) 50mM CaCl₂; and (**C**) 100 mM CaCl₂ upto 90 days.

The compressive strength (CS) results for the control and bio-cement mortar mixtures containing bacterial cells are presented in Fig. 5. The data clearly show a significant improvement in CS for all bacterial-treated samples compared with the control at 1, 7, 28, and 90 days. The overall enhancement in compressive strength upto 90 days can be attributed to the metabolic activity of the microbial cells within the cement–mortar matrix and the subsequent precipitation of nano-CaCO₃ via MICP. This biogenic CaCO₃ progressively seals microcracks, refines pore structure, and strengthens the cementitious network, thereby improving mechanical performance.

A notable observation is the marked increase in CS observed at 7 days compared with 90 days for all bio-cement mortars. During early curing (7 days), the porous structure of the fresh mortar provides sufficient space and moisture for microbial proliferation; however, the new, high-pH environment may initially restrict metabolic efficiency. At this stage, microbial cells rapidly precipitate calcite on their surfaces and within mortar pores, reducing porosity. This early-stage pore filling may inadvertently limit mass transport of nutrients and oxygen to the cells. Consequently, many microbes either die or transition into endospores, which may still contribute structurally by acting as organic micro-fillers, thereby supporting strength development.

These findings align with previous studies. Schwantes-Cezario et al.^44^ demonstrated that bacterial addition induces calcite precipitation that fills pore networks in cement mortars, reducing porosity and enhancing CS while lowering water absorption. Similarly, incorporation of *Sporosarcina pasteurii* improved CS by 22% and reduced water absorption by fourfold compared to untreated mortar^6^. In addition to the data in Supplementary Table 2, previously reported MICP bacteria generally yield compressive strength improvements of 15–45%, depending on nutrient composition and curing conditions. In contrast, *Bacillus albus* W39 achieved > 96% improvement under optimized conditions, underscoring its potential as a superior alternative to conventional strains such as *Sporosarcina pasteurii*.

The CS increased with bacterial concentration up to OD_600_ 1.5 at 25 mM CaCl₂ (mix B3-C25) (Fig. 5A). Specifically, the compressive strength of mix B3-C25 increased by 110.16%, 119.46%, 96.52%, 101.95%, and 102.05% at 1, 3, 7, 28, and 90 days, respectively, when compared with its corresponding control B0-C25 (Fig. 5A). However, increasing bacterial concentration from OD_600_ 0.5 to OD_600_ 1.5 at higher calcium concentrations (50 and 100 mM CaCl₂) resulted in a slight decline in CS relative to the control (Fig. 5B, C). This reduction may be attributed to excessive biomass accumulation or ionic stress caused by elevated calcium levels. High Ca²⁺ concentrations may impair bacterial metabolism, reduce urease activity, or trigger rapid, uncontrolled nucleation leading to agglomerated or poorly bonded CaCO₃ crystals. Such irregular deposits provide limited structural reinforcement and may disrupt microstructural cohesion^35^. The CS of mix B1-C50 increased by 117.99%, 120.53%, 135.63%, 123.09% and 166.35% whereas the CS of the B1-C100 mix increases.

with 178.64%, 157.60%, 192.40%, 185.68% and 183.51% than the CS of the corresponding control (B0-C50, B0-C100) for the pastes hydrated at 1,3,7,28, and 90days, respectively.

####  Bulk density

**Fig. 6:**
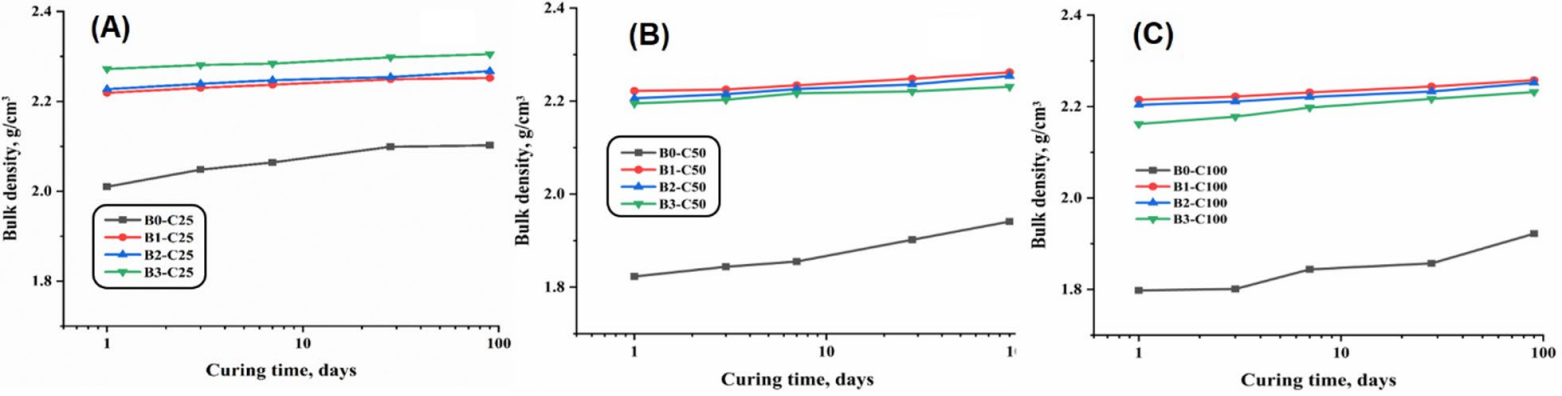
Bulk density of bio-mortar specimens treated with (**A**) 25 mM CaCl₂; (**B**) 50 mM CaCl₂; and (**C**) 100 mM CaCl₂ upto 90 days.

Bulk density (BD, g/cm³) serves as an important indicator of the internal compactness and porosity of cementitious materials, reflecting the efficiency of particle packing and the progression of hydration and mineralization processes. Higher BD values typically correspond to improved mechanical performance, as a denser matrix contains fewer voids and microcracks. As shown in Fig. 6A–C, all bacterial-treated samples exhibited a notable increase in BD over hydration periods of 1, 3, 7, 28, and 90 days. The highest BD values were recorded for mixes B3-C25, B1-C25, and B1-C100 when compared to the control at corresponding ages. This enhancement is attributed to biogenic calcite precipitation, which effectively fills pore spaces, reducing porosity and contributing to matrix densification^6^.

At 25 mM CaCl₂, the greatest BD value was observed for OD_600_ 1.5 (mix B3-C25), reflecting substantial calcite accumulation within the pore network and on bacterial cell surfaces. The bulk density enhancement for mix B3-C25 reached 13.03%, 11.38%, 10.66%, 9.48%, and 9.66% at 1, 3, 7, 28, and 90 days, respectively. In comparison, mixes B1-C50 and B1-C100 achieved corresponding increases of 21.89%, 20.66%, 20.43%, 18.19%, and 16.54%, and 23.19%, 23.38%, 20.99%, 20.84%, and 17.48% over the same curing ages.

This biomineralization effect narrows voids and strengthens particle interlocking, thereby increasing bulk density and compressive strength. Conversely, at higher CaCl₂ concentrations (50 and 100 mM), OD_600_ 0.5 yielded the highest BD values (mix B1-C50 and B1-C100). This reversal is likely due to osmotic stress induced by elevated calcium ion concentrations. High Ca²⁺ levels create a substantial osmotic gradient between the external solution and the bacterial cytoplasm, leading to cellular dehydration and reduced viability^45^. As a result, microbial urease secretion and metabolic activity decline, diminishing urea hydrolysis efficiency and limiting CaCO₃ precipitation. These effects collectively result in lower BD values at higher bacterial concentrations under elevated CaCl₂ conditions.

#### Hydration kinetics

**Fig. 7:**
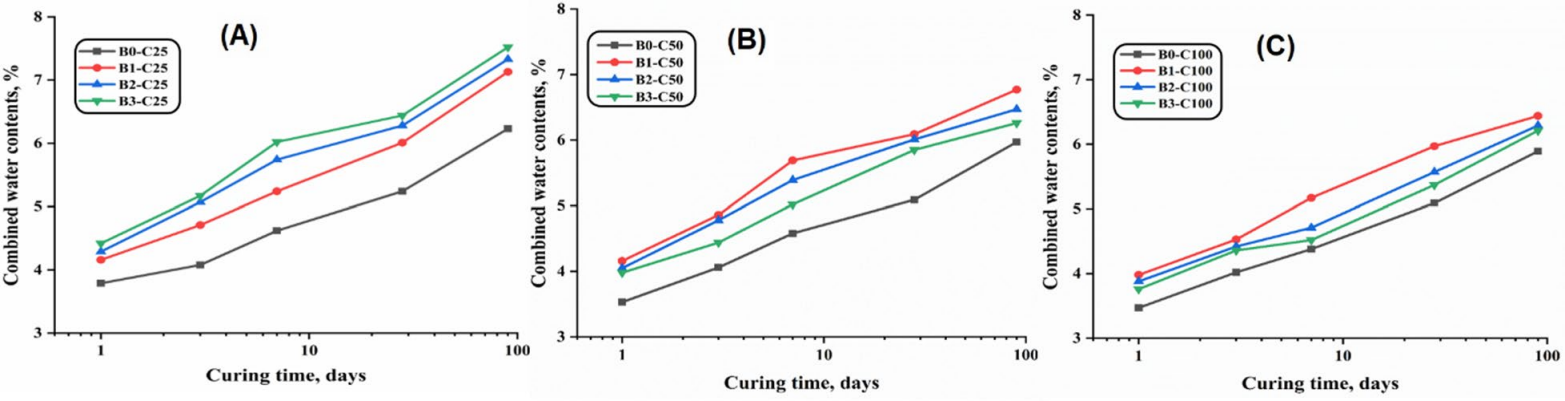
Chemically combined water of bio-mortar specimens treated with (**A**) 25 mM CaCl₂; (**B**) 50 mM CaCl₂; and (**C**) 100 mM CaCl₂ upto 90 days.

The hydration kinetics of the bio-modified mortar specimens were evaluated by measuring the chemically combined water content (Wn, %) over a curing period of up to 90 days, as shown in Fig. 7A–C. All bacteria-treated mixes displayed consistently higher Wn values than the untreated control throughout the hydration period. This behavior is associated with the nano-scale precipitation of CaCO₃ produced through MICP, which fills microcracks and capillary pores, thereby improving the mechanical performance of the cementitious matrix^45^. Beyond biomineralization, the gradual formation of hydration products also contributes to pore refinement, promoting increased compactness and densification of the mortar. Specimens cured in solutions containing 50 and 100 mM CaCl₂ (Fig. 7B, C) exhibited slightly lower Wn values than those cured at 25 mM CaCl₂ (Fig. 7A), indicating that excessive calcium availability may moderately inhibit hydration reactions or microbial efficiency. Despite these differences, bio-modified mortars at all bacterial concentrations (OD 0.5, 1.0, and 1.5) presented higher combined water contents than their respective controls. This improvement reflects the contribution of biogenic calcite, which occupies voids, enhances packing density, and increases the proportion of chemically bound water relative to untreated specimens.

The chemically combined water content increments for mix B3-C25 reached 17%, 27%, 30%, 23%, and 21% at 1, 3, 7, 28, and 90 days, respectively. In comparison, mixes B1-C50 and B1-C100 achieved corresponding increases of 13%, 9%, 10%, 15%, and 5%, and 8%, 9%, 3%, 6%, and 5% over the same curing ages. Because combined water content is a direct indicator of hydration progress, these increases are consistent with the observed enhancements in compressive strength and bulk density in the bio-modified mortars.

####  X-ray diffraction patterns

**Fig. 8:**
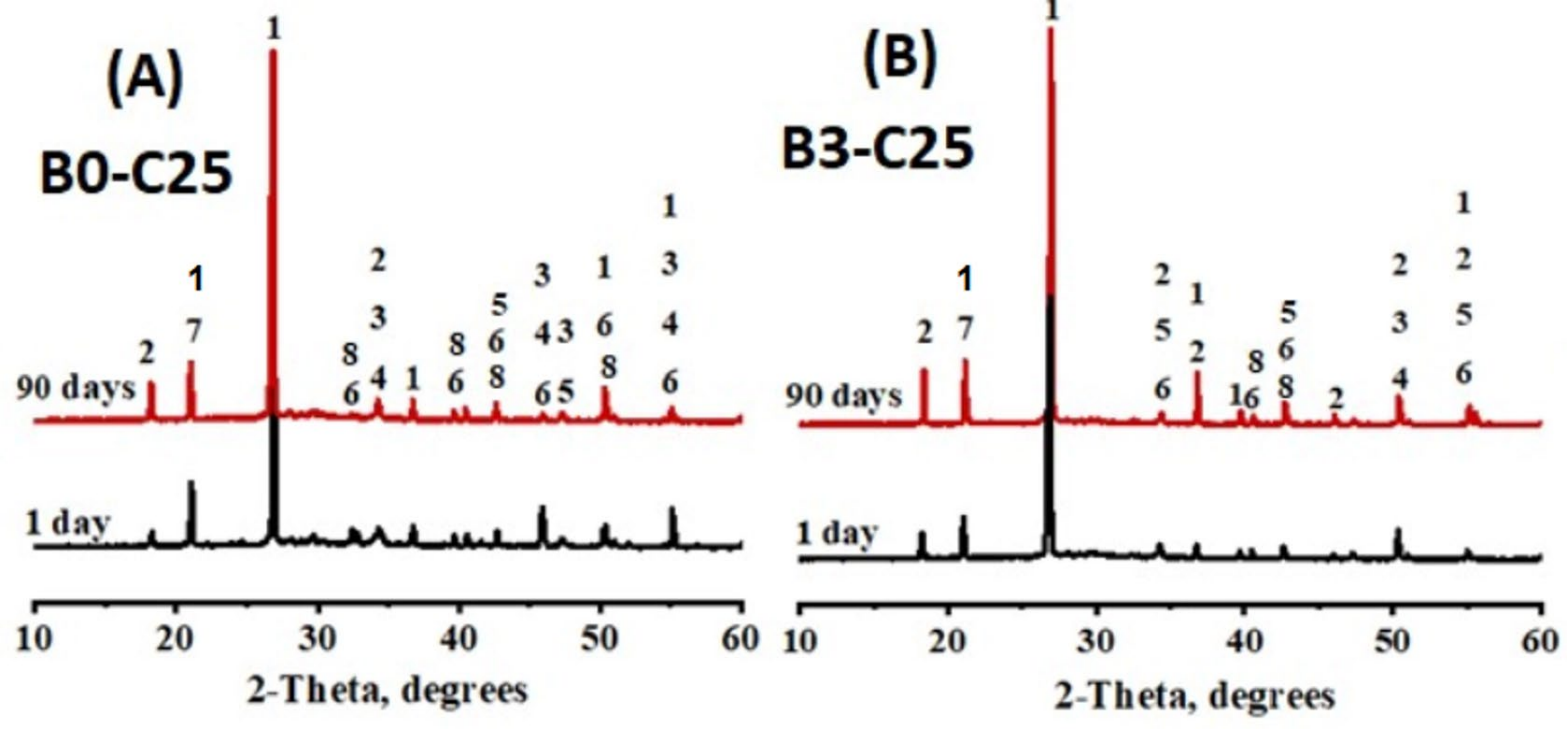
XRD-patterns for the selected mortar specimens; (**A**) B0-C25 and (**B**) B3-C25 cured at 1and 90 days; 1 = Quartz, 2 = CH, 3 = CSH, 4 = CASH, 5= $\:\mathrm{C}\stackrel{-}{\mathrm{C}}$, 6=C3S, 7=Ettringite, 8 = β-C2S.

**Fig. 9:**
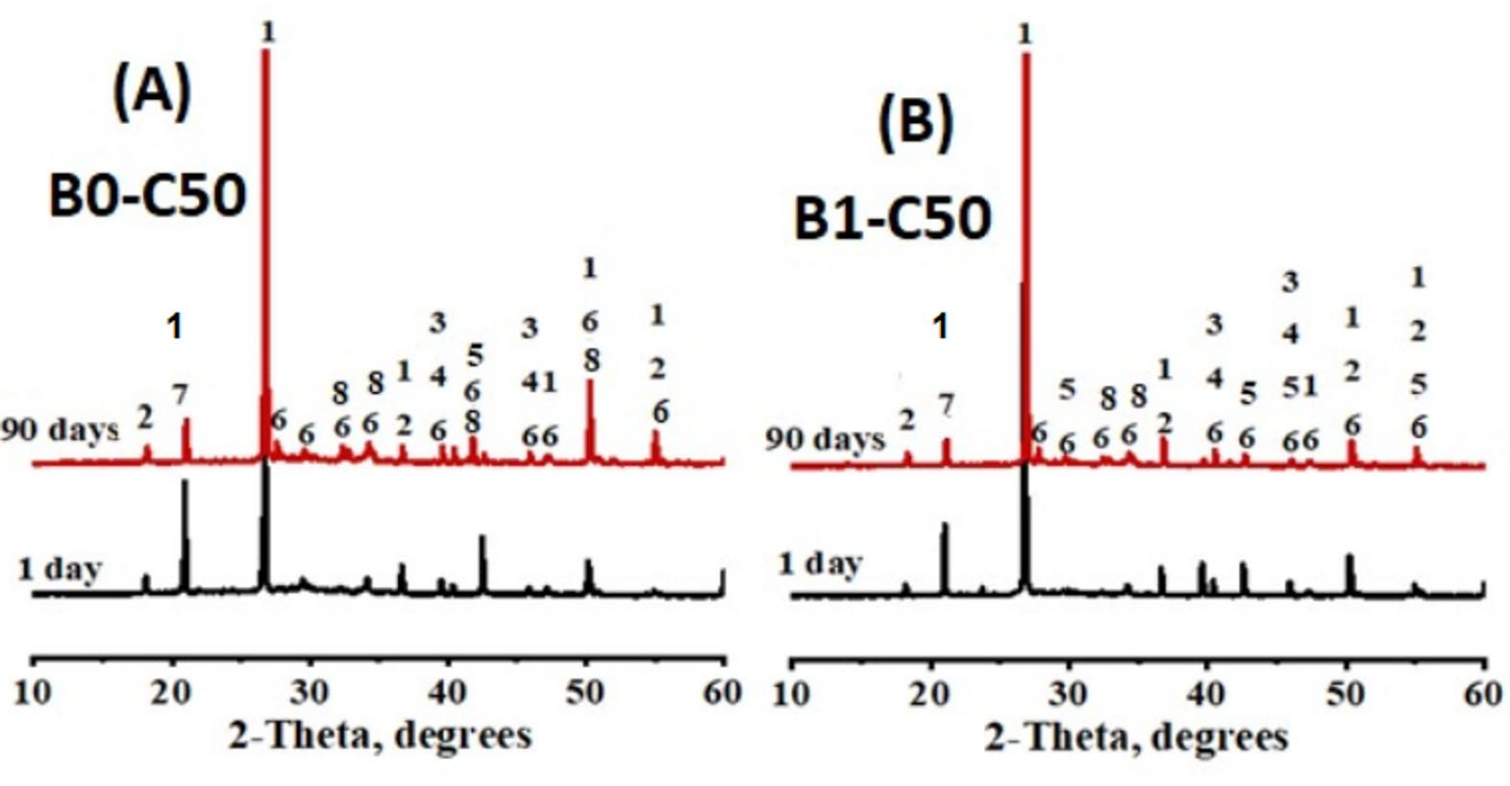
XRD-patterns of mortar specimens; (**A**) B0-C50, and (**B**) B1-C50 cured at 1 and 90 days; 1 = Quartz, 2 = CH, 3 = CSH, 4 = CASH, 5= $\:\mathrm{C}\stackrel{-}{\mathrm{C}}$, 6=C3S, 7=Ettringite, 8 = β-C2S.

**Fig. 10:**
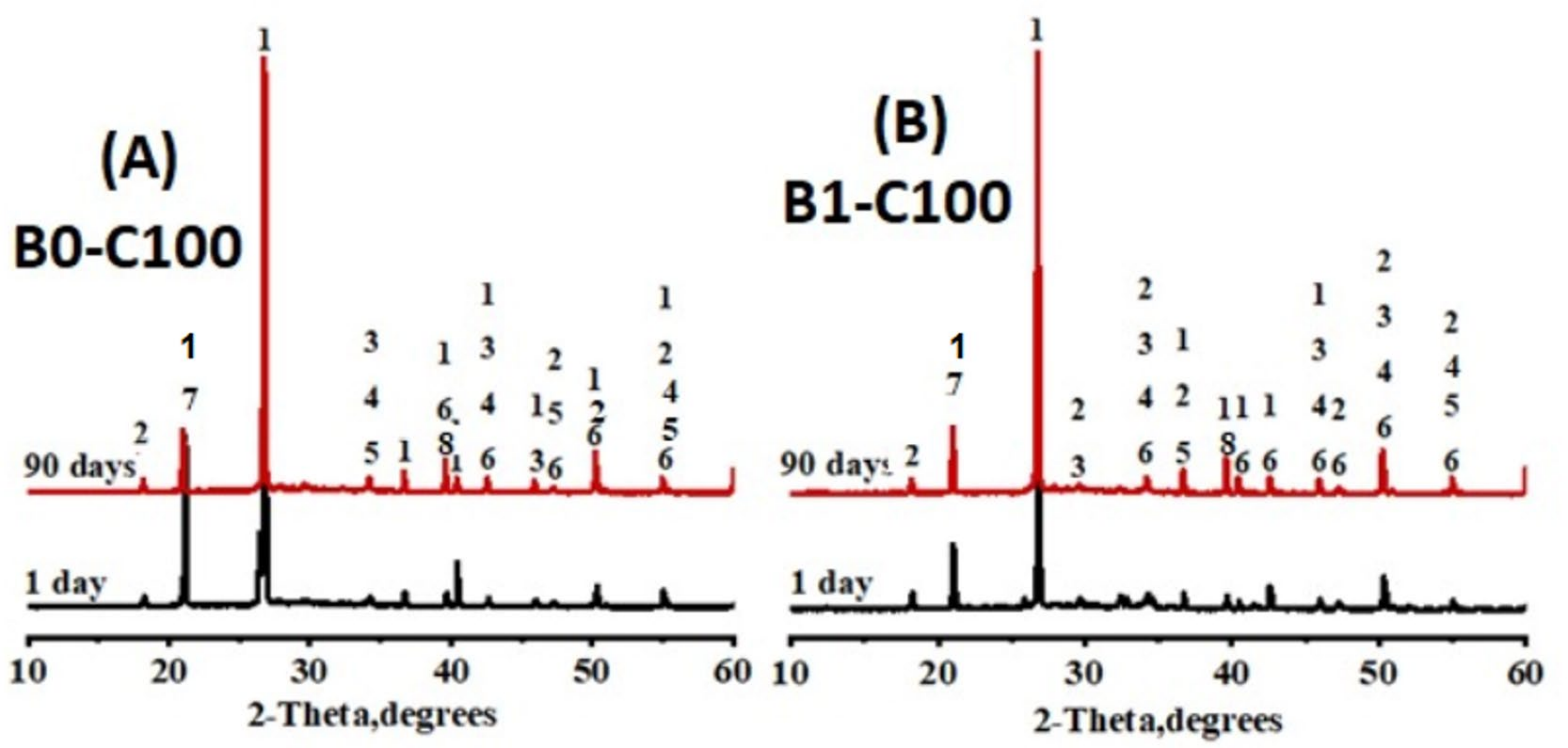
XRD-patterns of mortar; (**A**) B0-C100, and (**B**) B1-C100 cured at 1 day and 90 days of curing; 1 = Quartz, 2 = CH, 3 = CSH, 4 = CASH, 5= $\:\mathrm{C}\stackrel{-}{\mathrm{C}}$, 6=C3S, 7=Ettringite, 8 = β-C2S.

X-ray diffraction (XRD) is a widely used technique for characterizing crystallographic structures, phase composition, and material properties in cementitious systems. The XRD patterns of bio-incorporated mortars (B3-C25, B1-C50, and B1-C100) and their corresponding controls (B0-C25, B0-C50, and B0-C100) after 1 and 90 days of hydration are shown in Figs. 8, 9 and 10. The diffraction profiles revealed the presence of several crystalline phases. The most intense peak at 26.57° was assigned to quartz, indicating a substantial amount of unreacted sand, which is typical in cement–mortar formulations. A distinct peak at 17.97° corresponded to C–S–H, the principal hydration product responsible for the development of mechanical strength. Another peak at 20.79° was attributed to ettringite, formed during early hydration, and indicative of normal setting and hardening processes in cementitious materials.

Portlandite (CH) was identified at 34.03° and 47.05°, present in notable quantities as a by-product of cement hydration. These findings confirm the expected hydration sequence in OPC, with C–S–H, portlandite, and ettringite emerging as the dominant binding phases. The strong presence of portlandite and ettringite at 1 day reflects the early curing stage. Additional diffraction peaks at 2θ = 29.65°, 34.62°, 41.54°, 42.64°, 47.30°, 51.95°, 55.98°, and 56.72° were attributed to unreacted alite (3CaO·SiO₂), indicating that a portion of the clinker phase remained available for ongoing hydration.

In the bio-mortar samples containing *Bacillus albus* (W39), the XRD patterns are shown in Figs. 8, 9 and 10 at both 1 and 90 days, showing typical peaks for C–S–H and CH, in addition to newly formed calcite peaks at 2θ = 22.83°, 35.99°, 47.32°, 55.45°, and 60.21°. These calcite peaks were either absent or substantially weaker in the control samples, confirming microbial-induced CaCO₃ precipitation. Calcite formation was particularly prominent at 90 days, demonstrating sustained microbial activity and progressive biomineralization over time. A noticeable reduction in alite peak intensities was observed in the bio-mortars, indicating accelerated transformation of silicate phases into hydration products such as C–S–H and C–A–S–H. The presence of these phases contributes directly to the enhanced mechanical performance of the bio-modified mortar.

Overall, XRD analysis provides critical evidence linking microbial activity to hydration kinetics and mineral formation. These results offer valuable insights into the structural stability and long-term durability of bio-cement mortar^46^.

#### Thermal analysis

**Fig. 11:**
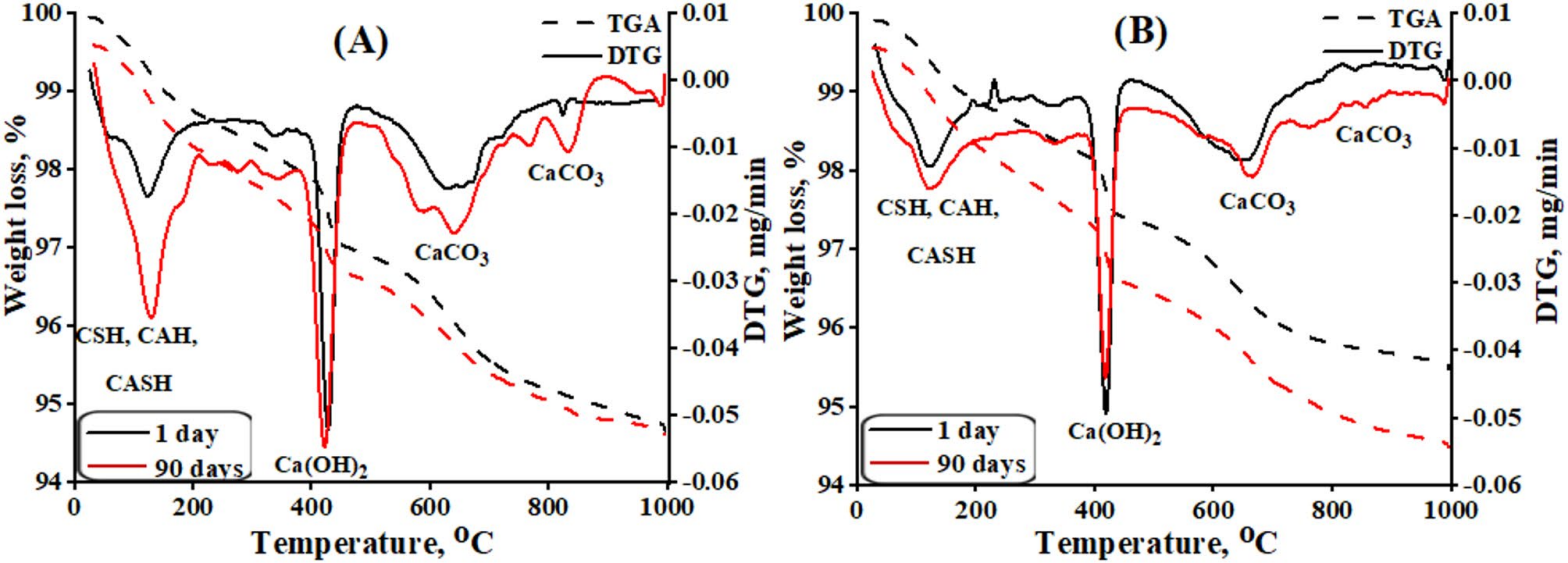
DTG/TGA curves for the selected mortar specimens (**A**) B0-C25, and (**B**) B3-C25 cured at 1 and 90 days.

**Fig. 12:**
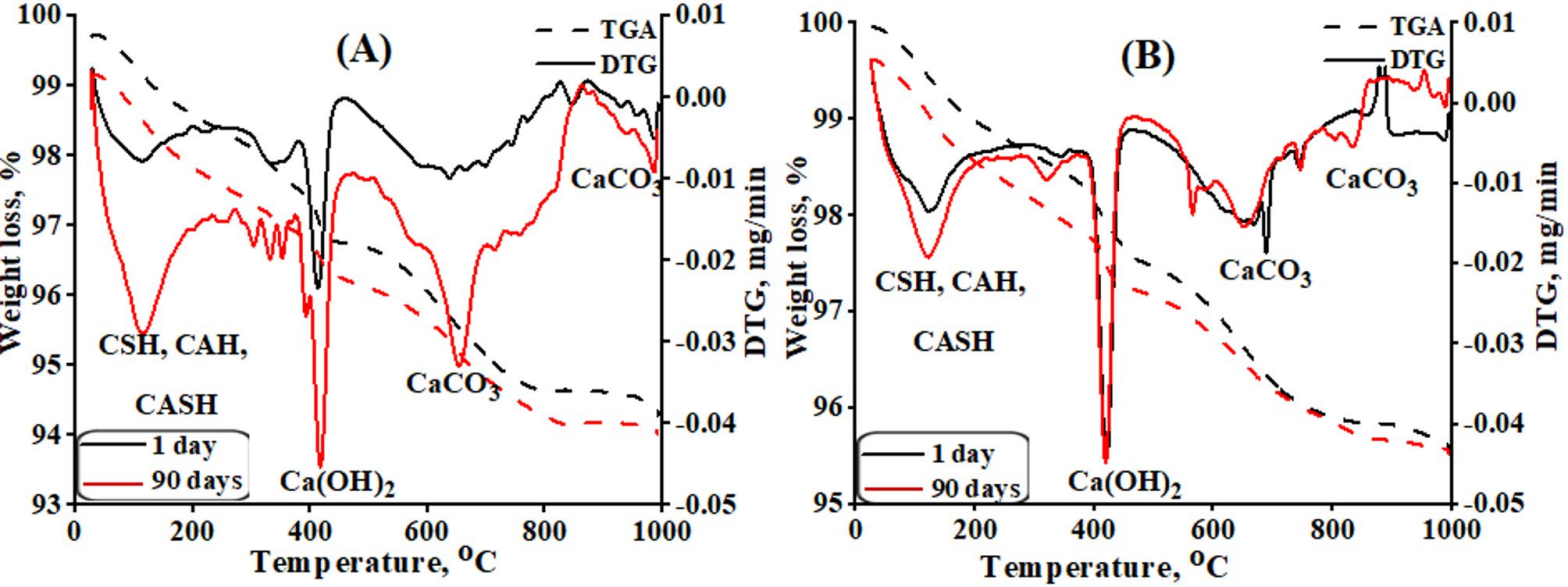
DTG/TGA curves for the selected mortar specimens (**A**) B0-C50, and (**B**) B1-C50 cured at 1 and 90 days.

**Fig. 13:**
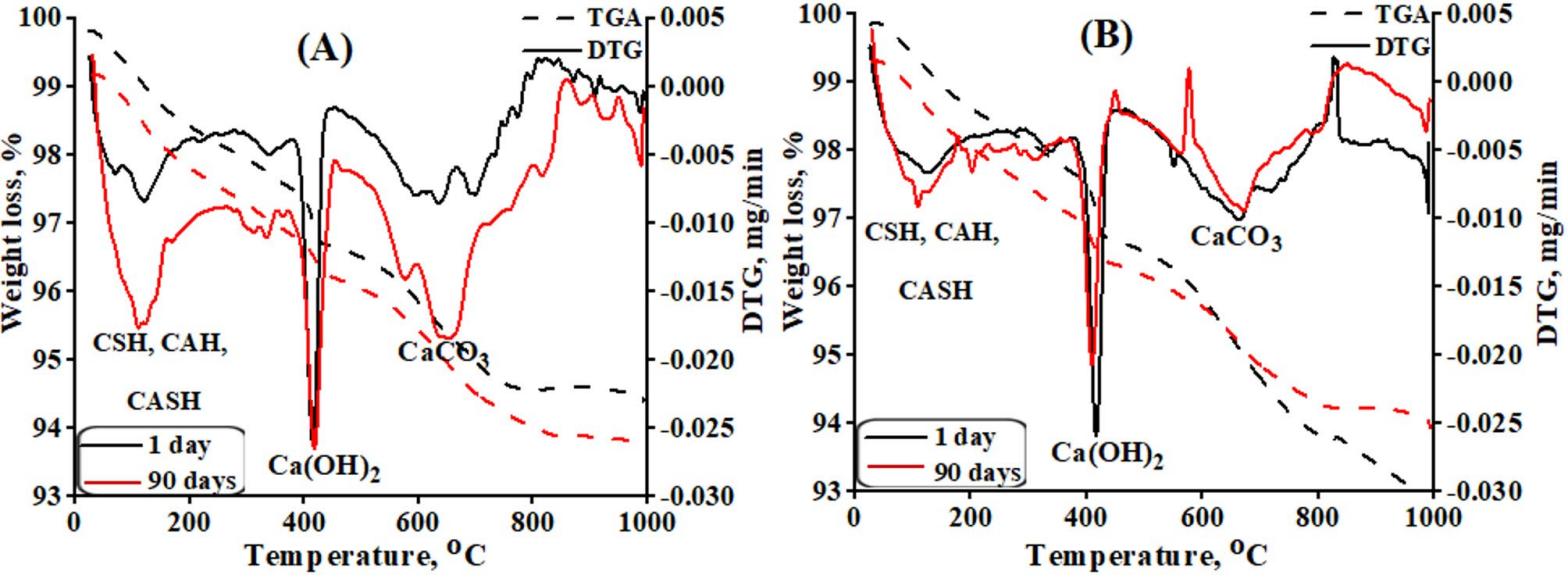
DTGA/TGA curves for the selected mortar specimens (**A**) B0-C100, and (**B**) B1-C100 at 1 and 90.

The DTA/TGA thermograms of the bio-modified mortar samples (B3-C25, B1-C50, and B1-C100) and the corresponding control mixtures (B0-C25, B0-C50, and B0-C100) after 1 and 90 days of curing are presented in Figs. 11, 12 and 13. The results revealed five distinct endothermic peaks. The first peak, observed between 58 and 65 °C, corresponds to the evaporation of physically adsorbed moisture in both control and bacterial specimens. The second endothermic peak, detected at 149–154 °C, is attributed to the evaporation of physically bound water and the dehydration or breakdown of amorphous hydration products, including C–S–H, C–A–S–H, and C–A–H. The enhanced intensity of this peak in the bacterial samples suggests improved cement hydration in the presence of microbial activity^6^.

A third endothermic peak was observed between 400 and 480 °C, corresponding to the thermal decomposition of portlandite (CH). In the range of 600–950 °C, endothermic peaks were associated with the decarbonation of amorphous and crystalline CaCO₃. When compared with the control, these decomposition peaks shifted to higher temperatures in the bacterial mortars, indicating an increase in both the quantity and crystallinity of biogenic calcium carbonate.

Among all mixes, sample B3-C25 (OD_600_ 1.5, and 25 mM CaCl₂) exhibited the most pronounced weight loss within the 50–200 °C range, indicating abundant formation of C–S–H, C–A–S–H, and C–A–H hydration products as shown in (Fig. 11B). The reduced CH weight loss in B3-C25 relative to the control suggests that a greater proportion of portlandite was converted into C–S–H and/or CaCO₃. Moreover, a higher weight loss above 600–800 °C in the bacterial samples reflects greater CaCO₃ decomposition, confirming effective MICP. The combined effects of increased C–S–H formation and enhanced biogenic CaCO₃ deposition in B3-C25 likely contribute to the superior compressive strength observed in this mix.

####  Morphology

**Fig. 14:**
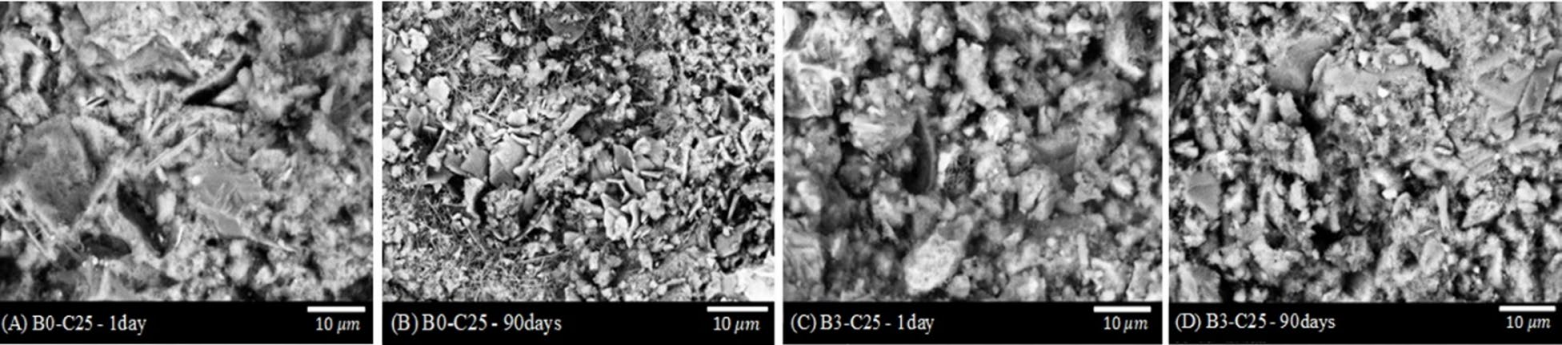
SEM micrographs of the selected motor specimens: B0-C25 at (**A**) 1 day and (**B**) 90 days of curing, and B3-C25 at (**C**) 1 day and (**D**) 90 days of curing.

**Fig. 15:**
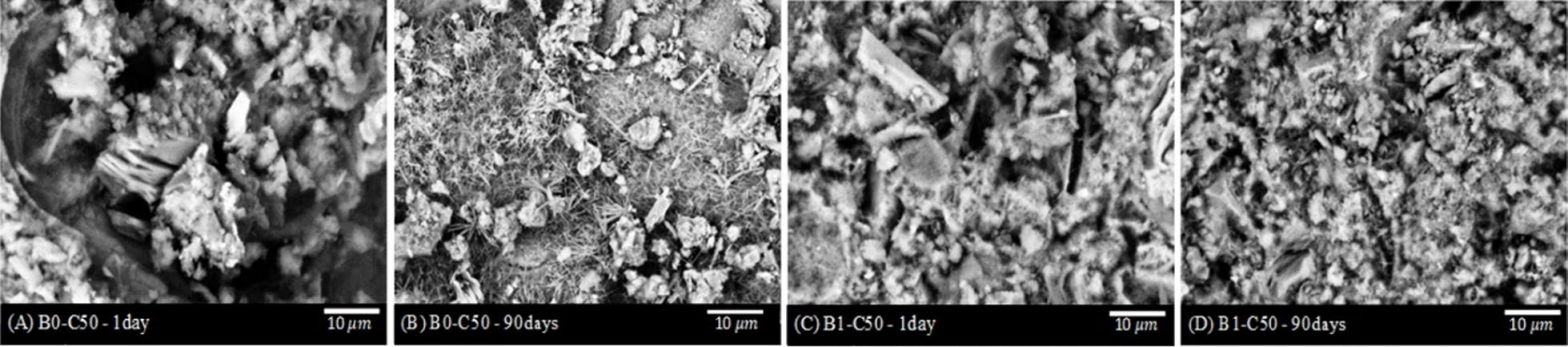
SEM micrographs of the selected motor specimens: B0-C50 at (**A**) 1 day and (**B**) 90 days of curing, and B1-C50 at (**C**) 1 day and (**D**) 90 days of curing.

**Fig. 16:**
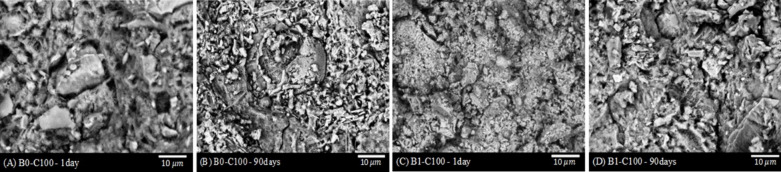
SEM micrographs of the selected motor specimens; B0-C100 at (**A**) 1 day and (**B**) 90 days of curing, and B1-C100 at (**C**) 1 day and (D) 90 days of curing.

The microstructure, surface morphology, and composition of the mortar specimens were investigated using SEM. Representative micrographs of the bio-modified mortars (B3-C25, B1-C50, and B1-C100) and their corresponding control mixtures (B0-C25, B0-C50, and B0-C100) after 1 and 90 days of curing are presented in Figs. 14, 15 and 16. The analysis aimed to visualize the formation and spatial distribution of CaCO₃ crystals within the matrix. The SEM images revealed abundant CaCO₃ precipitates filling the pore network of the bio-cement matrices, whereas the control specimens exhibited substantially fewer crystal deposits (Figs. 14 and 15 A, B). In the control mortars, CaCO₃ formation is primarily attributed to carbonation of portlandite (CH), a major hydration product of OPC, according to the reaction:

CO₂ + CH → CaCO₃ + H₂O.

However, carbonation in the untreated specimens remained limited due to the low availability of dissolved CO₂ in the pore solution. In contrast, the bio-cement mortars displayed a distinctive behaviour arising from the presence of bacterial cells and CaCl₂. The metabolic activity of the bacteria facilitated direct conversion of dissolved inorganic carbon into solid CaCO₃, while locally generated CO₂ reacted with CH, minimizing leaching and enhancing mineralization. This mechanism led to significantly greater CaCO₃ deposition, densifying the mortar structure and improving mechanical performance^47^. The incorporation of bacteria, therefore, not only enhances strength and durability but also contributes to lowering CO₂ emissions associated with cement production, supporting sustainable construction practice^6^. An increase in $\:\mathrm{C}\stackrel{-}{\mathrm{C}}$ content was observed with increasing bacterial concentration^48^. The SEM micrographs show that the bio-cement specimens were markedly denser and more compact, exhibiting reduced voids and microcracks compared with the untreated mixes (Figs. 14 and 15 C, D). Microbially induced calcium carbonate precipitation effectively decreases matrix permeability by filling pores and sealing microcracks, leading to improved structural integrity^12^. Nevertheless, elevated CaCl₂ concentrations (50 and 100 mM) may impose osmotic stress on bacterial cells, inhibiting growth and reducing biomineralization efficiency (Fig. 15 C, D). Furthermore, high CaCl₂ availability acts as a strong accelerator of cement hydration, which may cause rapid setting and potentially compromise long-term mechanical performance and durability.

Collectively, the microstructural evidence confirms that MICP, mediated by *Bacillus albus* (W39), induces substantial modifications in the mortar matrix, enhances hydration, and increases calcite deposition. These improvements translate into higher strength, reduced porosity, and superior durability, demonstrating the potential of this bio-based technology for developing resilient, self-healing bio-concrete.

### Durability of bio-cement mortar against 5%-MgSO_4_ and 5%-MgCl_2_ attack solutions

#### Compressive strength (CS)

**Fig. 17:**
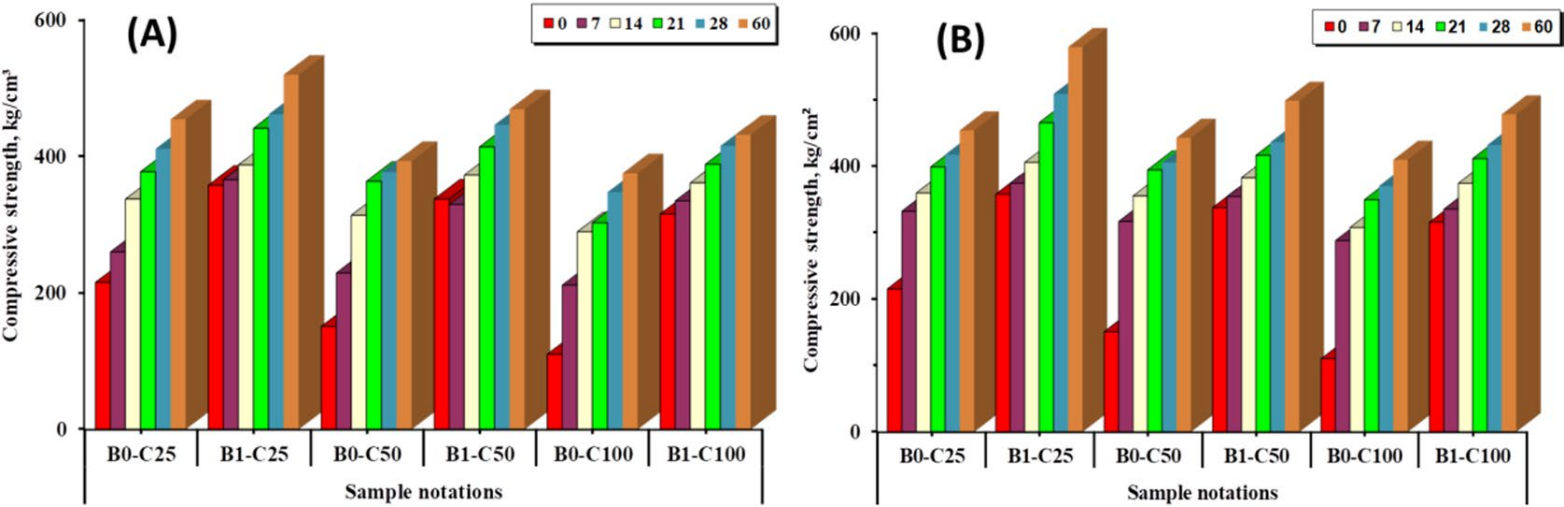
The compressive strength values of the bio-cement and control mortar samples immersed in (**A**) 5% MgSO₄ or (**B**) 5% MgCl₂ solution upto two months.

The compressive strength (CS) of the bio-modified mortar specimens (B1-C25, B1-C50, and B1-C100) and their corresponding controls (B0-C25, B0-C50, and B0-C100), after immersion in 5% MgSO₄ or 5% MgCl₂ solutions for two months, is shown in Fig. 17A, B. All mixes exhibited a progressive increase in CS during the two-month curing period, with the bio-mortars consistently achieving higher strength values than the untreated specimens. This enhancement is attributed to the combined effects of MICP and cement hydration, where biogenic CaCO₃ crystals provide additional nucleation sites between sand and cement particles, supporting continuous strength gain during curing. Simultaneously, the progressive pore refinement and crack sealing hinder the ingress of sulphate ions (SO₄²⁻), delaying brucite formation, mitigating C–S–H decalcification, and limiting the development of expansive ettringite. Consequently, biogenic mineralization contributes to sustained compressive strength, reduced porosity, and improved resistance against magnesium sulphate attack^49,50^.

Among all evaluated mixes, B1-C25 exhibited the greatest resistance to MgSO₄ deterioration, maintaining consistently higher CS values than B0-C25 at all curing ages. Specifically, the compressive strength of B1-C25 increased by 40.75%, 14.83%, 16.89%, and 12.27% at 7, 14, 28, and 60 days, respectively, when compared with its corresponding control (Fig. 17A). Under MgCl₂ exposure, the observed strength enhancement was associated with accelerated cement hydration, where Mg²⁺ ions penetrated the matrix and adsorbed onto C–S–H surfaces, promoting crystallization and microstructural densification (Fig. 17B). However, CS values declined as CaCl₂ concentration increased from 25 mM to 50 and 100 mM, suggesting adverse effects of excessive calcium.

Calcium ions are essential for MICP; high CaCl₂ concentrations increase the ionic strength and osmotic pressure, which can suppress bacterial metabolism and existence, resulting in reduced biogenic CaCO₃ formation. This reduction lowers pore-filling efficiency and reduces mechanical improvements. Furthermore, CaCl₂ may react with calcium hydroxide (Ca(OH)₂) to form calcium oxychloride (Ca(OCl)₂), a phase known to degrade concrete strength^51^. In MgCl₂ solution, the CS of B1-C25 increased by 12.66%, 12.81%, 16.66%, and 21.89% at 7, 14, 28, and 60 days, respectively, compared with B0-C25 (Fig. 17B).

The durability assessment confirms that the incorporation of *Bacillus albus* (W39) markedly improves the resistance of mortars exposed to destructive chemical environments. The bio-modified mixtures exhibited higher compressive strength than the control specimens when immersed in both MgSO₄ and MgCl₂ solutions. This improvement arises from the combined action of MICP and cement hydration, where biogenic CaCO₃ precipitates seal microcracks and refine the pore network structure, thereby lowering permeability and restricting the penetration of aggressive ions. As a result, sulphate-induced decalcification, brucite formation, and ettringite or gypsum expansion were diminished, leading to sustained mechanical stability. The greatest enhancement was recorded for specimens prepared at OD₆₀₀ = 0.5 and 25 mM CaCl₂ (B1-C25), indicating that this composition provided optimal conditions for MICP activity and CaCO₃ nucleation. In contrast, higher CaCl₂ concentrations (50–100 mM) resulted in lower strength gains, likely due to osmotic stress and reduced bacterial viability. The potential formation of calcium oxychloride at elevated chloride contents may also have contributed to a decline in material integrity. Under MgCl₂ exposure, strength improvements were further supported by Mg²⁺ adsorption on C–S–H phases, which accelerated hydration and enhanced crystallization within the matrix. The durability results show that MICP plays a vital role in improving structural performance by promoting closed-matrix densification, sustaining higher compressive strength, and limiting microstructural degradation under chemically aggressive attack. These findings provide evidence that bio-cement approaches can produce durable, eco-efficient construction materials capable of maintaining long-term performance in harsh service environments.

#### Characteristics of pore structure

**Fig. 18:**
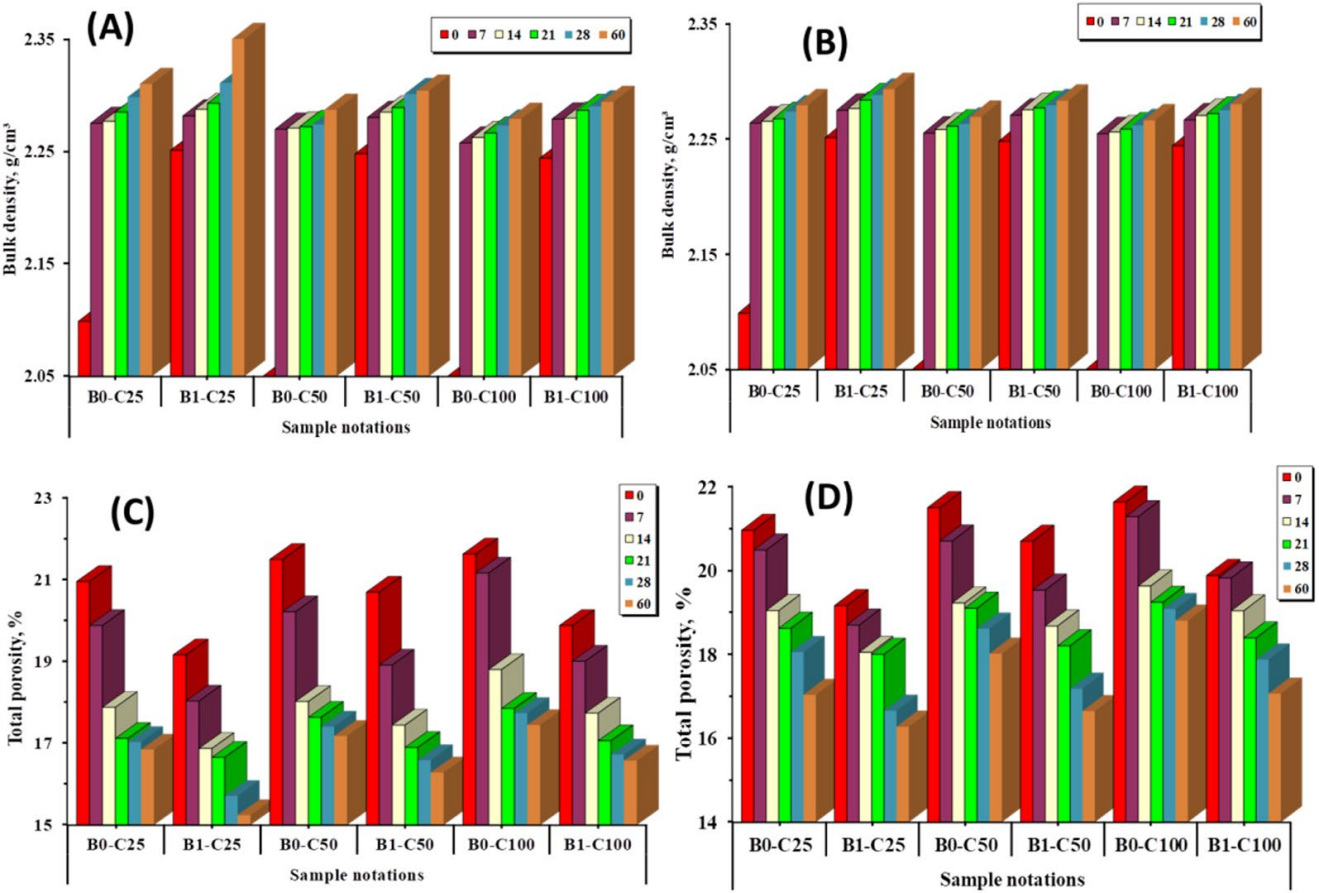
Bulk density and total porosity of the bio-cement and control mortar samples immersed in (**A**&**C**) 5% MgSO₄ or (**B**&** D**) 5% MgCl2 solution upto two months.

To clarify the physico-mechanical behavior of the produced mortar specimens, their pore structure was assessed through bulk density (BD, g/cm³) and total porosity (TP, %). The BD and TP values of bacterial-modified mortars immersed in 5% MgSO₄ and 5% MgCl₂ solutions for upto two months are shown in Fig. 18. As illustrated in Fig. 18A, B, all bio-cement mortars exhibited a progressive rise in BD throughout the curing period, consistent with the expected trend of matrix densification over time. Correspondingly, Fig. 18C, D shows a decrease in TP for the same mixes, confirming microstructural refinement. The BD values of bio-cement mixes (B1-C25, B1-C50, and B1-C100) were consistently higher than those of the corresponding control specimens (B0-C25, B0-C50, and B0-C100). This enhancement is attributed to the continuous formation of hydration products and biogenic CaCO₃, which efficiently fill internal voids, reducing porosity and increasing resistance to the ingress of MgSO₄ and MgCl₂ ions.

In addition, biogenic calcite crystals provide nucleation sites for further growth of C–S–H and C–A–S–H phases, yielding a more compact matrix with lower porosity. This self-induced mineralization not only increases bulk density but also decreases total porosity, thereby limiting ion transport and improving durability. The improved BD and reduced TP values provide clear evidence of the protective role of MICP against sulphate- and chloride-induced deterioration^52^.

Among all the investigated mixes, B1-C25 displayed the most pronounced improvement, achieving the lowest porosity and highest bulk density at 60 days. After 60 days in MgSO₄ (Fig. 18A), BD increased by 3.25%, 0.74%, and 0.66% for B1-C25, B1-C50, and B1-C100, respectively, while TP decreased by 9.5%, 5.2%, and 4.9%, respectively (Fig. 18B). In MgCl₂ exposure, the B1-C25 mix also showed sustained improvement, with BD increasing by 0.53%, 0.49%, 0.75%, 0.62%, and 0.61% after 7, 14, 21, 28, and 60 days of immersion, respectively. Correspondingly, total porosity declined by 8.70%, 5.21%, 3.32%, 7.73%, 8.22%, and 18.57% at the same immersion durations (Fig. 18D).

#### Total sulphate (TS) and total chloride (TC) contents

**Fig. 19:**
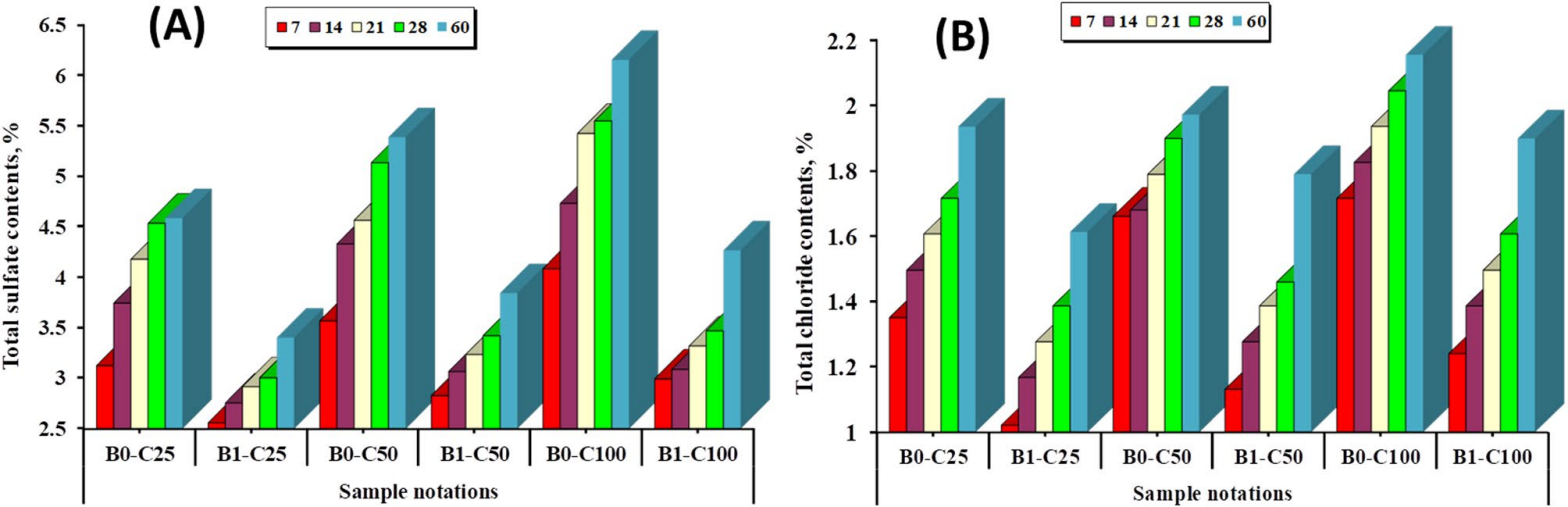
The TS (**A**) and TC (**B**) contents of the bio-cement and control mortar samples immersed in (**A**&**C**) 5% MgSO₄ or (**B**&** D**) 5% MgCl2 solution upto two months.

Figure 19A, B presents the total sulphate (TS) and total chloride (TC) contents for bio-mortars immersed in 5% MgSO₄ or 5% MgCl₂ solutions for up to two months. In all specimens, TS and TC values increased progressively with longer immersion time, reflecting continued ion penetration during exposure. However, the bio-modified mortars (B1-C25, B1-C50, and B1-C100) consistently exhibited lower TS and TC contents than their corresponding controls (B0-C25, B0-C50, and B0-C100). This reduction is attributed to MICP, which refines the pore structure, decreases permeability, and inhibits the ingress of aggressive SO₄²⁻ and Cl⁻ ions. The improved resistance against chemical attack is consistent with the sealing of microcracks and densification of the matrix through MICP.

An increase in ion entrance was observed as the CaCl₂ concentration increased from 25 mM to 50 mM and 100 mM, indicating that excessive calcium concentrations diminish bacterial activity and reduce the efficiency of pore filling. After two months of immersion in MgSO₄, the TS contents of the bio-cement mortars (B1-C25, B1-C50, and B1-C100) were reduced by 25.71%, 28.59%, and 30.66%, respectively, compared to the control specimens (Fig. 19A). Similarly, after two months in MgCl₂ solution, the TC values for the bio-mortars were 3.40%, 3.85%, and 4.27%, respectively. In contrast, the control mortars exhibited higher TC values of 4.59%, 5.39%, and 6.15% under identical exposure conditions (Fig. 19B).

These results confirm that reduced TS and TC contents are directly associated with higher chemical resistance. The findings highlight the positive role of MICP in enhancing the durability of bio-mortars, particularly in the mix B1-C25, which consistently demonstrated the lowest ion ingress and the highest durability^46,53^.

#### X-ray diffraction patterns

**Fig. 20:**
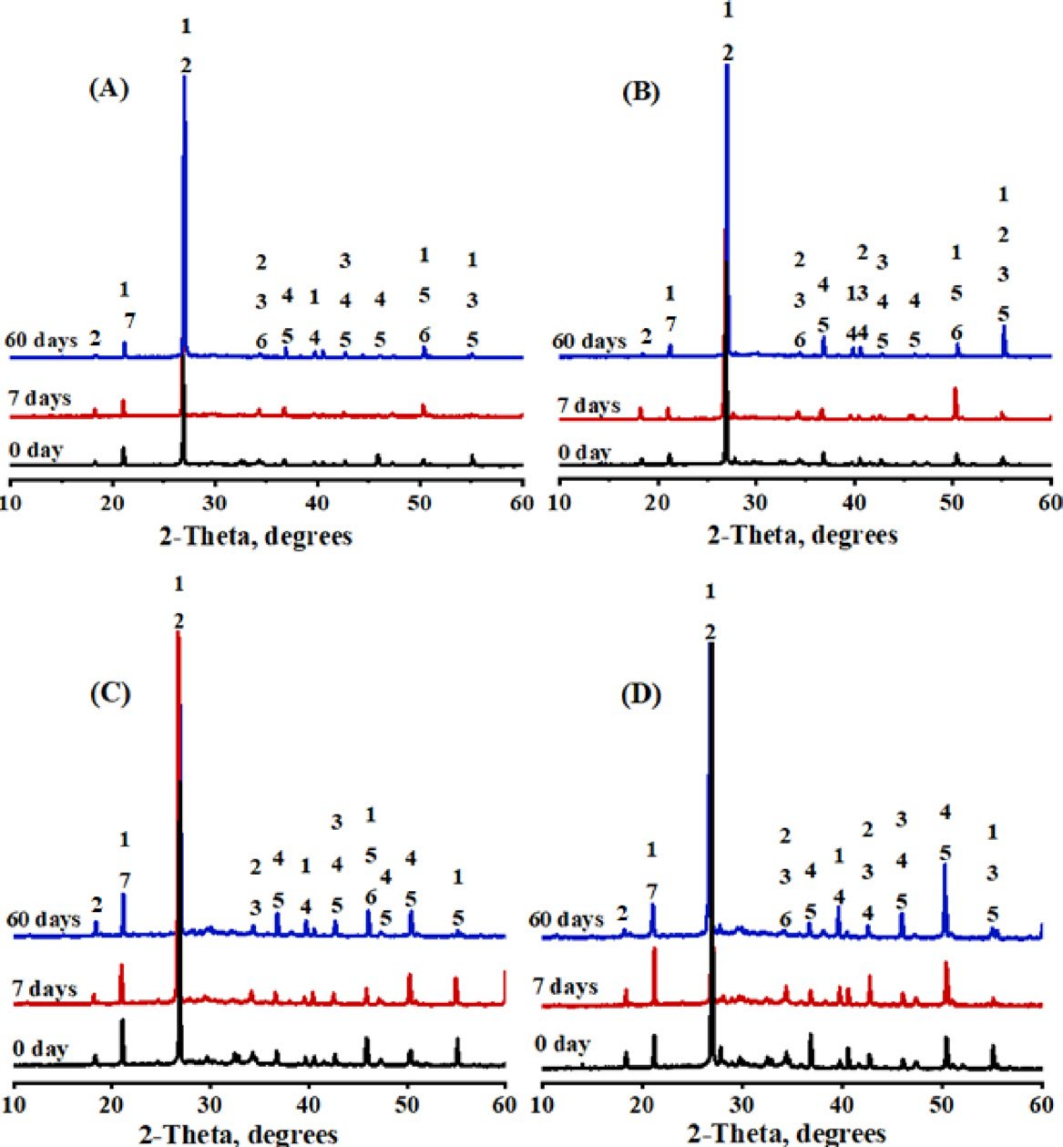
XRD patterns of B0-C25 (**A** &** C**) and B1-C50 (**B** &**D**) mixes immersed in 5% MgSO4 or MgCl2 for 0, 7 and 60 days, 1 = Quartz, 2 = CH, 3 = CSH, 4= $\:\mathrm{C}\stackrel{-}{\mathrm{C}}$, 5 = β-C2S, 6=C3S, 7=Ettringite.

The XRD patterns of the B0-C25 and B1-C25 specimens immersed in 5% MgSO₄ or 5% MgCl₂ solutions for two months are presented in Fig. 20A–D. The diffraction profiles confirm the presence of typical cement hydration products, including C–S–H, CH, C$\:\stackrel{-}{\mathrm{C}}$, β-C₂S, C₃S, and quartz. As expected, a reduction in the intensities of the anhydrous β-C₂S and C₃S phases was observed with extended curing, indicating the progressive development of hydration reactions over time. In the control specimens (B0-C25), distinct diffraction peaks corresponding to brucite (Mg(OH)₂) were detected following exposure to MgSO₄ or MgCl₂ solutions (Fig. 20A–C). Brucite formation occurs when CH reacts with Mg²⁺ ions under aggressive attack. In MgSO₄ environments, this process results in the production of Mg(OH)₂, while the simultaneous formation of CaSO₄·2 H₂O promotes secondary reactions with calcium–aluminate hydrates to yield expansive ettringite^54^. This mechanism consists of the diffraction patterns of the control specimens in Fig. 20A, B. Similarly, immersion in MgCl₂ solution leads to brucite formation through the interaction of CH with MgCl₂, producing Mg(OH)₂ and CaCl₂. The latter subsequently reacts with calcium aluminate phases to form Friedel’s salt (C₃A·CaCl₂·10 H₂O), as illustrated in Fig. 20C, D^17^. Both deterioration mechanisms promote pore expansion, microcracking, and destabilization of C–S–H.

In contrast, the bio-modified specimens (B1-C25) exhibited markedly reduced brucite peak intensities and additional reflections associated with CaCO₃ at both curing ages, confirming the effectiveness of MICP. The absence of Mg(OH)₂ peaks in the B1-C25 patterns (Fig. 20B, D) indicates that biogenic CaCO₃ consumed available CH, suppressing brucite formation and limiting subsequent interactions with Mg²⁺, SO₄²⁻, and Cl⁻ ions. This mineralogical stabilization explains the superior durability of the bacterial cement mortars under both SO₄²⁻ and Cl⁻ ions attack.

#### Scanning electron microscopy

**Fig. 21:**
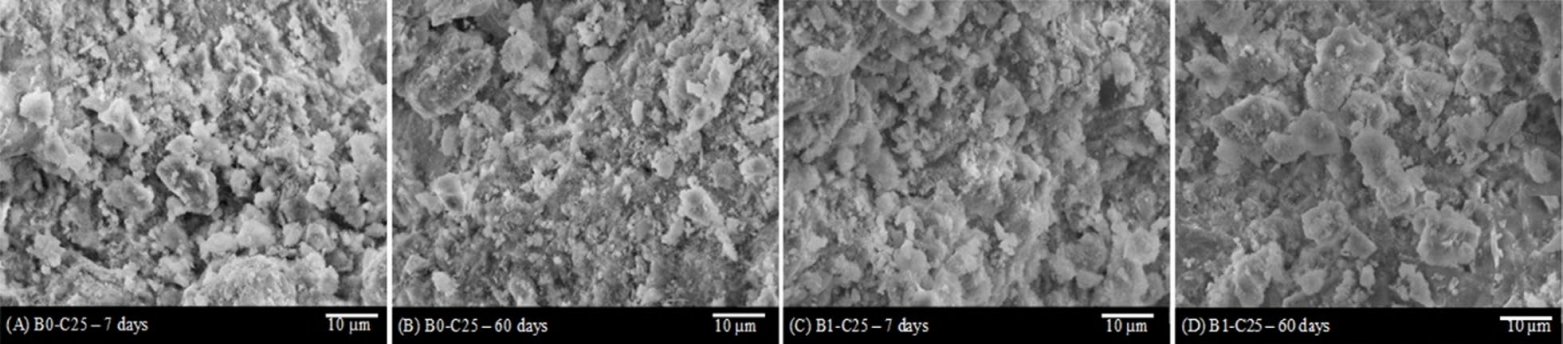
SEM micrographs of the selected mixes submerged in 5% MgSO_4_; (**A**) B0-C25 at 7 days, (**B**) B0-C25 at 60 days, (**C**) B1-C25 at 7 days, and (**D**) B1-C25 at 60 days.

**Fig. 22:**
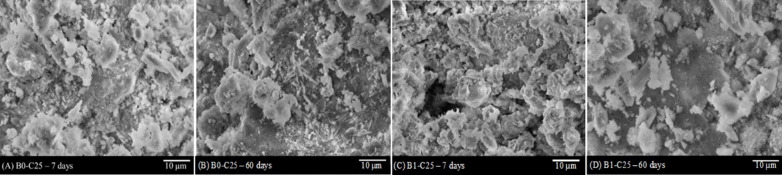
SEM micrographs of the selected mixes submerged in 5% MgCl_2_; (**A**) B0-C25 at 7 days, (**B**) B0-C25 at 60 days, (**C**) B1-C25 at 7 days, and (**D**) B1-C25 at 60 days.

The SEM micrographs of B0-C25 and B1-C25 specimens immersed in 5% MgSO₄ or 5% MgCl₂ for 60 days are shown in Figs. 21A–D and 22A–D, respectively. After 7 days of exposure in MgSO₄, the control mortar (B0-C25) exhibited a relatively porous microstructure, characterized by plate-like Ca(OH)₂ crystals and poorly crystallized C–S–H gel, along with early ettringite formation (Fig. 21A). These features indicate an open pore network that facilitates sulphate ingress, thereby weakening the matrix. After 60 days, the microstructure of B0-C25 showed clear signs of deterioration, including numerous needle-like ettringite and secondary gypsum crystals that disrupted matrix continuity. These expansive products increased sulphate uptake and contributed to reduced compressive strength (Fig. 21B).

The corresponding micrographs of the bio-cement mortar (B1-C25) immersed in MgSO₄ are presented in Fig. 21C, D. At 7 days, a thin initial layer of bacterial-induced CaCO₃ was observed in coincidence with hydration products at the surface (Fig. 21C). This layer partially refined the pore network and increased compactness relative to the control. After 60 days, the B1-C25 microstructure exhibited a markedly denser matrix, with extensive bacterial CaCO₃ deposition interlocked with micro- and nanocrystalline hydration phases (Fig. 21D). This compact microstructure effectively restricted the penetration of Mg²⁺ and SO₄²⁻ ions, suppressing the formation of expansive degradation products and enhancing long-term durability.

Figure 22A–D presents the micrographs of B0-C25 and B1-C25 specimens exposed to MgCl₂. At 7 days, B0-C25 displayed a flocculent and porous texture, with poorly crystalline C–S–H embedded within large capillary pores (Fig. 22A). This morphology provided pathways for chloride ingress. After 60 days, the control mortar revealed abundant hexagonal Friedel’s salt plates (3CaO·Al₂O₃·CaCl₂·10 H₂O) occupying the pore structure, indicative of extensive chloride penetration and binding (Fig. 22B)^55^.

In contrast, the bio-cement mortar (B1-C25) showed significant biogenic CaCO₃ precipitation and additional C–S–H formation after 7 days in MgCl₂ (Fig. 22C), resulting in a largely closed matrix with improved compressive strength. After 60 days, only limited deposits of Friedel’s salt were observed within the bio-mortar (Fig. 22D). The interlocking crystalline networks formed by bacterial CaCO₃ and C–S–H reduced pore connectivity and restricted chloride transport. These observations demonstrate that bacterial incorporation not only refined the microstructure but also minimized the extent of chloride-binding phases, further enhancing durability.

### Effect of elevated temperature on bio-cement mortar

#### Compressive strength

**Fig. 23:**
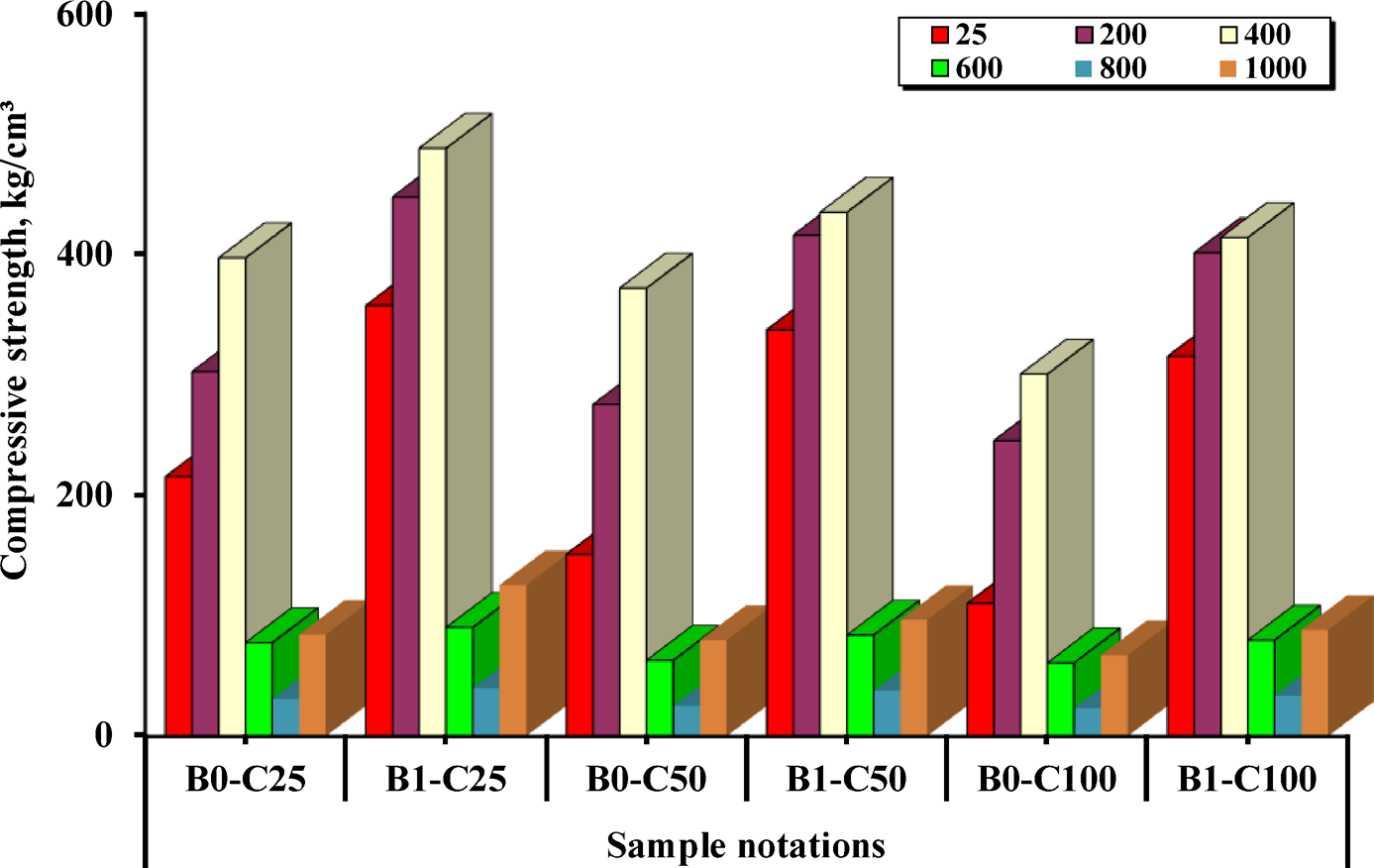
Compressive strength of bio-cement mortars thermally treated upto 1000 °C.

Thermal stability of the mortar specimens was assessed after 28 days of hardening, followed by exposure to progressively elevated temperatures (200, 400, 600, 800, and 1000 °C). The results, presented in Fig. 23, demonstrate that all bio-cement mortars exhibited a similar thermal response. A notable increase in CS was observed at 400 °C, attributed to internal autoclaving and hydrothermal reactions within the pore network during heating. This process promotes the hydration of previously unreacted cement phases, leading to the formation of additional thermally stable hydrates, including C–S–H, C–A–S–H, and C–A–H. The development of these phases enhances matrix integrity and strength, consistent with the TGA/DTGA and XRD findings^56^.

A marked decline in CS was observed between 600 and 800 °C due to the progressive decomposition of hydration products and the formation of β-C₂S. These reactions increase total porosity, resulting in a more open pore structure and reduced mechanical performance. Interestingly, at 1000 °C, a slight increase in CS was recorded. This improvement may be associated with densification and recrystallization of newly formed phases at high temperatures, producing a more rigid and consolidated structure^57,58^.

Overall, bacterial cement mortars exhibited superior thermal resistance compared to control specimens. This enhancement is primarily attributed to MICP and the generation of additional hydrates, which fill pore spaces and produce a denser, more interlocked microstructure. The bio-treated specimens demonstrated noticeably better performance at elevated temperatures, as CaCO₃ precipitation contributes to matrix densification and self-healing. The greater availability of lime particles further improves self-repair capacity and thermal stability^59^.

####  Ignition loss

**Fig. 24:**
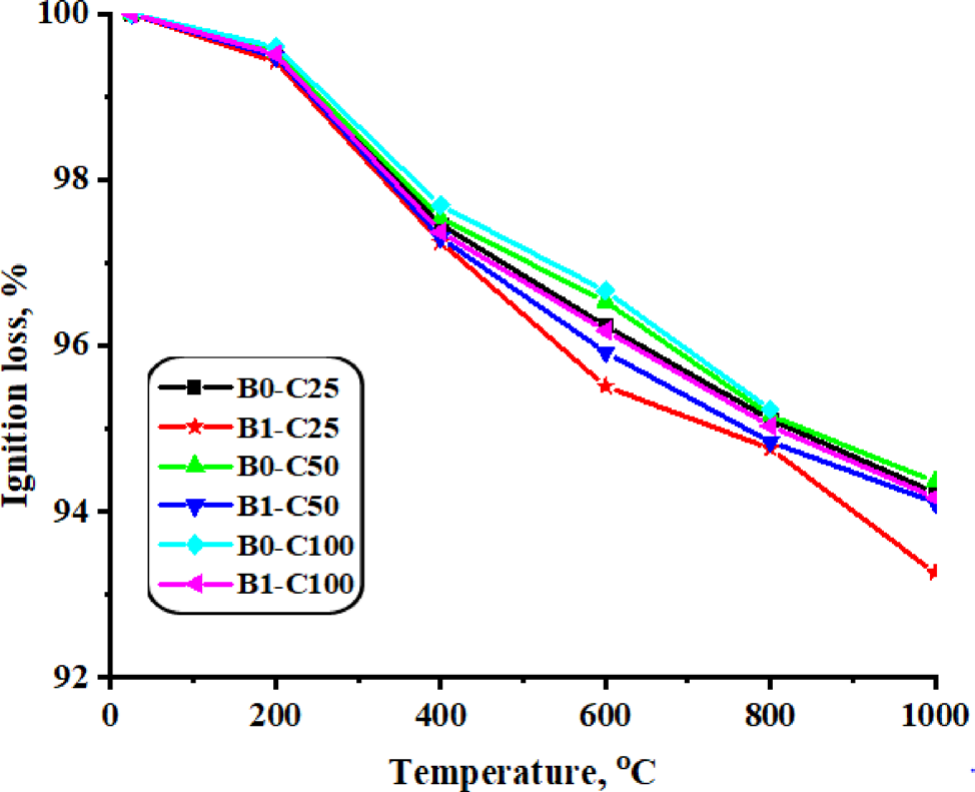
Ignition loss of bio-cement mortars thermally treated upto 1000 °C.

As shown in Fig. 24, weight loss in the mortar specimens increased progressively with rising temperature, reflecting the thermal decomposition of hydration products. Initial dehydration occurred at approximately 105 °C, followed by partial decomposition of several hydrates—including C–S–H, C–A–H, C–A–S–H, and sulfoaluminate hydrates (AFm and AFt)—around 200 °C. At temperatures above 450 °C, CH decomposition was evident, while the decarbonation of $\:\mathrm{C}\stackrel{-}{\mathrm{C}}$ occurred within the 650–750 °C range.

The incorporation of bacterial cells resulted in noticeably higher weight loss up to 1000 °C compared to the control specimens. This behavior can be attributed to greater consumption of liberated portlandite and the formation of additional hydration products, which filled internal pore spaces and generated a compact, interlocking nanostructure^60^. The increased formation of thermally sensitive hydrates and biogenic $\:\mathrm{C}\stackrel{-}{\mathrm{C}}$ explains the enhanced thermal stability observed in the bio-modified mortars.

#### X-ray diffraction patterns

**Fig. 25:**
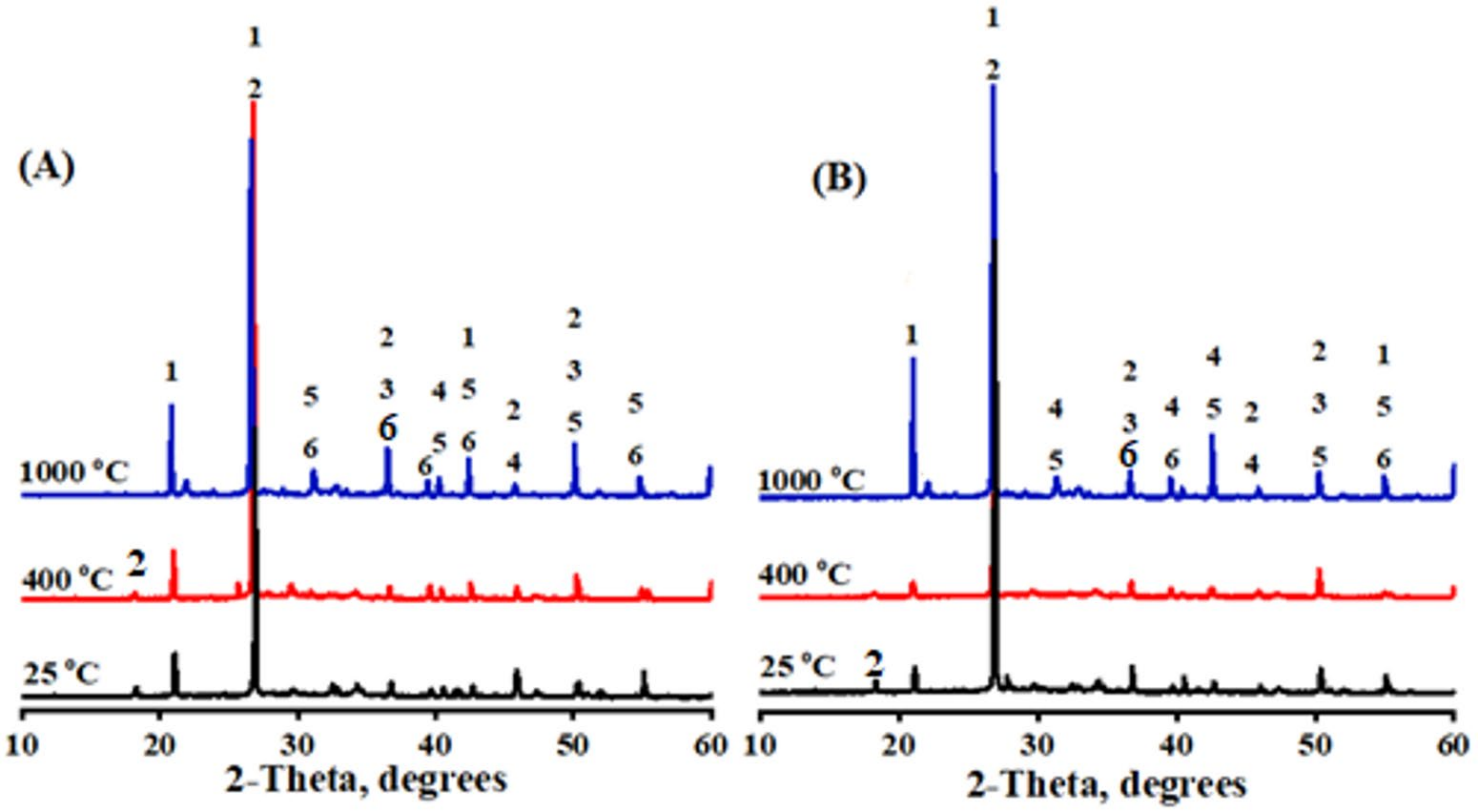
The XRD patterns of the bio-cement mortars (**A**) B0-C25, (**B**) B1-C25 thermally treated upto 1000 °C; 1 = Quartz, 2 = CH, 3 = CSH, 4= $\:\mathrm{C}\stackrel{-}{\mathrm{C}}$, 5 = β-C_2_S, 6=C_3_S.

Figure 25A, B shows the XRD patterns of the fired bio-cement mortar samples (B0-C25 and B1-C25) at elevated temperatures up to 1000 °C. In the hydrated state, the dominant phases were identified as C–S–H gel, portlandite (CH), and CaCO₃, along with residual unhydrated clinker minerals (C₃S and β-C₂S). When the temperature increased to 400 °C, a noticeable intensification of the diffraction peaks associated with CH and C–S–H was observed. This behavior can be attributed to hydrothermal reactions and internal autoclaving, which promote further hydration of unreacted C₃S and β-C₂S phases.

With further heating, the intensity of CH peaks gradually decreased, whereas the diffraction lines corresponding to C–S–H became more prominent. At 600 °C, the reduction in CH peak intensity became more pronounced, reflecting partial decomposition and transformation into CaO. At 1000 °C, the diffraction peak of CaCO₃ disappeared, indicating complete thermal degradation and conversion to CaO. Concurrently, the C–S–H phases were fully eliminated and transformed into thermally stable crystalline phases, predominantly β-C₂S and C₃S.

#### Differential/thermogravimetric analysis (DTG /TGA)

**Fig. 26:**
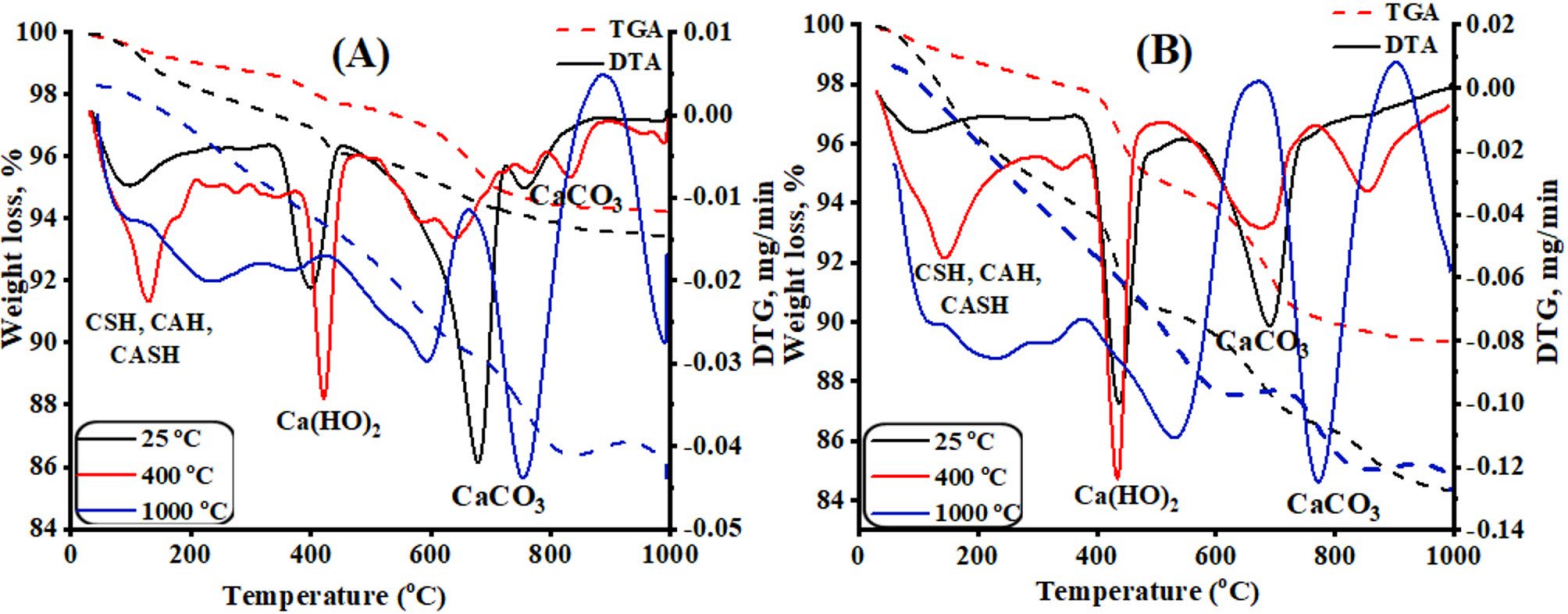
DTG/TGA curves of the bio-cement mortars (**A**) B0-C25, (**B**) B1-C25 thermally treated upto 1000 °C.

Figure 26A, B shows the DTG/TGA curves of the fired bio-cement mortars (B0-C25 and B1-C25) subjected to thermal treatment upto 1000 °C. Four distinct endothermic peaks were identified within the ranges of 85–220 °C, 410–500 °C, 605–660 °C, and 810–970 °C. The first peak (85–220 °C) is associated with the evaporation of physically adsorbed water and the dehydration of amorphous hydration products, namely C–S–H, C–A–H, and C–A–S–H. The second peak, at approximately 410–500 °C, corresponds to the dehydroxylation of portlandite (CH). The third and fourth peaks, detected at around 650 °C and 750 °C, are attributed to the decomposition of amorphous and crystalline CaCO₃, respectively^61^.

At 400 °C, the mass losses associated with the first endothermic reaction (< 200 °C) were 0.66% for B0-C25 and 1.01% for B1-C25. These results indicate an increase in hydration products in the bio-modified mortar, reflecting the beneficial influence of bacterial incorporation. This improvement is ascribed to MICP, which provides additional nucleation sites for the formation of C–S–H, C–A–H, and C–A–S–H. Similarly, the mass losses corresponding to CH decomposition were 1.23% for B0-C25 and 1.45% for B1-C25, demonstrating enhanced carbonation and greater CH consumption when bacteria were present. In addition to accelerating hydration, MICP contributes to CH depletion through secondary reactions, resulting in a denser microstructure.

A notable increase in mass loss between 600 and 950 °C further confirms the presence of biogenic CaCO₃, as this temperature interval corresponds to carbonate decomposition. These effects were more pronounced, highlighting the sustained influence of microbial activity. At 1000 °C, both mortars showed substantial reductions in hydration products due to complete CH decomposition and the disappearance of C–S–H. The total mass losses were 0.22% for B0-C25 and 0.34% for B1-C25. Although the bacterial-treated specimens exhibited higher weight loss in the CaCO₃ decomposition region (600–950 °C), their microstructure remained less prone to thermal collapse, supporting the lasting advantage of bacterial incorporation in improving high-temperature resistance.

The thermal analysis confirms that mortars incorporating *Bacillus albus* display enhanced mechanical stability across a wide temperature range. The initial increase in CS at 400 °C is attributed to hydrothermal reactions that hydrate residual clinker phases and generate additional thermally stable compounds. With increasing temperature, decomposition of C–S–H and portlandite proceeds; however, MICP-derived CaCO₃ contributes to matrix densification and delays microstructural degradation. DTG/TGA results show greater weight losses in bio-treated mortars, particularly within the CaCO₃ decomposition range, reflecting a higher carbonate content. Despite this, the bio-modified specimens retained better microstructural integrity at elevated temperatures, which is attributed to the denser, interlocking matrix produced through bacterial mineralization. At 1000 °C, both mixes underwent extensive phase decomposition and recrystallization, yet the bacterial mortars showed superior residual stability, confirming the long-term benefit of MICP in improving thermal resistance. Overall, these findings demonstrate that microbial incorporation promotes the formation of additional hydrates and CaCO₃, refines the pore system, and produces a more robust and durable mortar capable of withstanding severe thermal exposure.

Future Work and Recommendations.

This study focused on mechanical and durability improvements of bio-mortars with *Bacillus albus* W39, without direct crack-healing visualization or quantification. In addition, the urea-enriched CaCl₂ curing system, while effective in the lab, raises concerns about ammonia release and field applicability. Future studies should therefore investigate alternative metabolic pathways, including non-ureolytic biomineralization systems, encapsulated nutrient delivery, or low-urea formulations to minimize ammonia release while maintaining effective self-healing performance.

Further optimization of the relationship between bacterial concentration, nutrient availability, and curing conditions is required, particularly for large‑scale applications where environmental variability may affect MICP performance. The use of alternative calcium sources and nutrient carriers should be examined to reduce cost and environmental impact while maintaining consistent calcite precipitation and durability.

Field‑scale evaluation of *Bacillus albus* W39 under fluctuating temperature, humidity, and exposure to aggressive media is recommended. Scaling up bacterial production, storage, and delivery remains a practical constraint; therefore, immobilization or encapsulation methods should be explored to improve bacterial survivability and controlled release in concrete.

Incorporation of MICP with supplementary cementitious materials such as fly ash, slag, metakaolin, or geopolymers may further enhance mechanical and environmental performance. These studies will support the development of cost‑effective, resilient, and sustainable bio‑based construction materials for future infrastructure.

## Conclusions

This study demonstrates the potential of the alkaliphilic *Bacillus albus* strain W39, isolated from Wadi El-Natrun, to enhance cementitious materials through microbially induced calcium carbonate precipitation (MICP). Under optimized conditions (pH 8, 25 mM CaCl₂, 20 g/L urea, 30 °C, 7 days), the strain produced up to 0.453 g/100 mL of nanoscale calcite, confirmed by instrumental analyses. Incorporation of viable bacterial cells into mortar improved compressive strength during 90 days of curing, with performance dependent on the balance between bacterial concentration and calcium ion availability. The most effective outcome was observed at OD₆₀₀ = 1.5 with 25 mM CaCl₂, while OD₆₀₀ = 0.5 was more suitable at higher Ca²⁺ concentrations (50–100 mM).

Microstructural characterization (XRD, TGA/DTG, SEM) revealed reduced porosity and a denser matrix due to biogenic calcite precipitation and additional C–S–H formation. These changes contributed to improved durability, including resistance to MgSO₄ and MgCl₂ attack, and enhanced thermal stability up to 1000 °C. Despite these promising findings, the study has limitations. Experiments were conducted under controlled laboratory conditions, and long-term field validation is required to confirm performance in real service environments. The durability tests focused on selected parameters, and further assessments such as freeze–thaw cycling and additional chemical exposures are recommended. Moreover, scalability, cost-effectiveness, and ecological impacts of microbial incorporation remain to be evaluated.

Within these limits, *Bacillus albus* W39 emerges as a promising candidate for sustainable self-healing concrete, offering measurable improvements in strength, durability, and resilience while reducing reliance on conventional repair methods.

## Supplementary Information

Below is the link to the electronic supplementary material.

Supplementary Material 1

## Data Availability

All data generated or analysed during this study are included in this published article and its Supplementary Information files. Additional raw datasets are available from the corresponding authors upon reasonable request.
