# Supplementary material for: Microbially induced calcite precipitation by a novel alkaliphilic Bacillus albus strain for sustainable self-healing bio-mortar with enhanced mechanical performance and durability
Source: Sci Rep. 2026 Jun 23;16:19549. doi: 10.1038/s41598-026-57485-3 (PMC13291362; doi:10.1038/s41598-026-57485-3)
Supplement: Supplementary file 1 — Supplementary Material 1 [file 41598_2026_57485_MOESM1_ESM.docx]

**Microbially Induced Calcite Precipitation by a Novel Alkaliphilic Bacillus albus Strain for Sustainable Self-Healing Bio-Mortar with Enhanced Mechanical Performance and Durability**

Sahar. M. Ibrahim^1^, Dalia Said^2^, Mohamed Heikal^1^, Mohamed O. Abdel-Monem^2^, Ghada E. Dawwam^2^

^1^Chemistry Department, Faculty of Science, Benha University, Benha, Egypt.

^2^Botany and Microbiology Department, Faculty of Science, Benha University, Benha, Egypt.

* Corresponding authors:

*E-mail addresses:* mohamed.heikal@fsc.bu.edu.eg (M. Heikal), sahar.abdelhameed@fsc.bu.edu.eg (S.M. Ibrahim).

**Supplementary Table 1**: Amount of precipitated CaCO_3_ using B4 broth media.

| **Isolate code** | **Soil Sample** | | **Final pH** | **Absorbance**  **(**$\boldsymbol{\lambda}$**=600 nm)** | **CaCO_3_** **(g/100ml)** | **Isolate code** | **Soil Sample** | **Final pH** | **Absorbance**  **(**$\boldsymbol{\lambda}$**=600 nm)** | | **CaCO_3_ (g/100ml)** |
| --- | --- | --- | --- | --- | --- | --- | --- | --- | --- | --- | --- |
| W1 | **1^st^**  soil sample | | 8.89 | + | 0.057 | W26 | **3^rd^**  soil sample | 9.03 | + | | 0.056 |
| W2 |  |  | 8.91 | ++ | 0.200 | W27 |  | 9.00 | + | | 0.093 |
| W3 |  |  | 8.69 | ++ | 0.250 | W28 |  | 9.06 | ++ | | 0.197 |
| W4 |  |  | 8.62 | + | 0.043 | W29 | **4^th^**  soil sample | 6.65 | + | | 0.043 |
| W5 |  |  | 8.63 | + | 0.083 | W30 |  | 6.07 | + | | 0.003 |
| W6 |  |  | 8.94 | ++ | 0.293 | W31 |  | 8.81 | +++ | | 0.307 |
| W7 |  |  | 8.85 | + | 0.130 | W32 |  | 9.05 | + | | 0.120 |
| W8 |  |  | 8.95 | + | 0.060 | W33 |  | 6.52 | + | | 0.017 |
| W9 | **2^nd^**  soil sample | | 8.83 | + | 0.077 | W34 |  | 8.88 | ++ | | 0.290 |
| W10 |  |  | 8.87 | + | 0.040 | W35 |  | 8.89 | + | | 0.063 |
| W11 |  |  | 8.83 | + | 0.057 | W36 |  | 6.45 | +* | | 0.013 |
| W12 |  |  | 8.75 | + | 0.047 | W37 |  | 8.89 | + | | 0.070 |
| W13 |  |  | 8.59 | + | 0.067 | W38 |  | 8.36 | + | | 0.120 |
| W14 |  |  | 8.90 | + | 0.153 | W39 |  | **9.21** | **+++** | | **0.453** |
| W15 |  |  | 8.88 | ++ | 0.213 | W40 |  | 8.63 | + | | 0.057 |
| W16 |  |  | 8.97 | + | 0.053 | W41 |  | 8.73 | + | | 0.073 |
| W17 |  |  | 8.81 | + | 0.093 | W42 |  | 8.68 | + | | 0.057 |
| W18 |  |  | 8.70 | + | 0.056 | W43 | **5^th^**  soil sample | 8.97 | ++ | | 0.170 |
| W19 |  |  | 8.85 | + | 0.060 | W44 |  | 8.77 | + | | 0.030 |
| W20 |  |  | 8.71 | + | 0.043 | W45 |  | 8.71 | + | | 0.043 |
| W21 | **3^rd^**  soil sample | | 9.05 | +++ | 0.313 | W46 |  | 8.73 | + | | 0.087 |
| W22 |  |  | 9.02 | ++ | 0.286 | W47 |  | 8.87 | ++ | | 0.207 |
| W23 |  |  | 8.92 | + | 0.103 | W48 |  | 8.76 | + | | 0.053 |
| W24 |  |  | 9.03 | ++ | 0.233 | W49 |  | 8.75 | ++ | | 0.180 |
| W25 |  |  | 9.06 | ++ | 0.170 | W50 |  | 8.94 | + | | 0.060 |
|  | | *** + low, ++ moderate, +++ high growth.** | | | | | | | |  | |

**Supplementary Table 2: Comparative performance of *Bacillus albus* and commonly studied ureolytic bacteria in bio‑concrete applications.**

| **Bacteria** | **Mechanism** | **Nutrient System** | **Compressive Strength ↑ (%)** | **Reference** |
| --- | --- | --- | --- | --- |
| *Sporosarcina pasteurii* | Ureolysis | Lactose Mother Liquor waste water, Urea, Nutrient broth, Yeast Extract | ∼17% | [1] |
| *Sporosarcina pasteurii* |  | Urea, Yeast Extract | ∼33% | [2] |
| *Sporosarcina pasteurii* |  | Urea, Yeast Extract, CaCl₂ | ∼35% | [3] |
| *Bacillus sphaericus* |  | Urea, Yeast Extract, Calcium Nitrate | >40% | [4] |
| *Bacillus cereus* |  | Tofu waste water, Nutrient Broth | ∼27.8% | [5] |
| *Enterobacter sp.* |  | Lentil seeds, Sugar, Urea, Beef extract | ∼23% | [6] |
| *Bacillus subtilis* |  | Peptone, Yeast extract | 16.6% | [7] |
| ***Bacillus albus* (B3-C25)**  **(This study)** |  | Yeast extract + Urea + CaCl₂ | >96% | This study |

**Morphological and Biochemical Characterization of Bacterial Isolates**


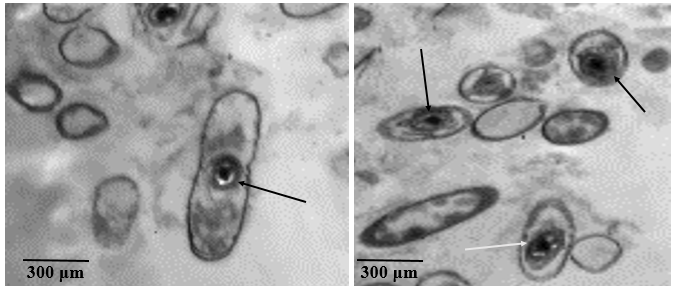
Supplementary Figure 1: TEM images of Bacillus albus spores

**3.5. Characterization of Precipitated CaCO_3_**

**3.5.1. TEM and SEM/EDX Characterization**

The nanoscale morphology and dispersion of precipitated CaCO_3_ nanoparticles were examined using high-resolution transmission electron microscopy (HR-TEM), Supplementary Fig. 2**,** and field emission scanning electron microscopy (FE-SEM), Supplementary Fig.3. As illustrated in Supplementary Figure 2, the CaCO₃ particles exhibit a rhombohedral crystalline morphology, characteristic of calcite commonly observed in biogenic mineralization. These rhombohedral structures appear aggregated into spherical and semi-spherical shapes. TEM analysis further revealed that the particle size ranged from 50 to 65 nm, indicating nanoscale precipitation and suggesting a biologically mediated self-assembly mechanism during the crystallization process.


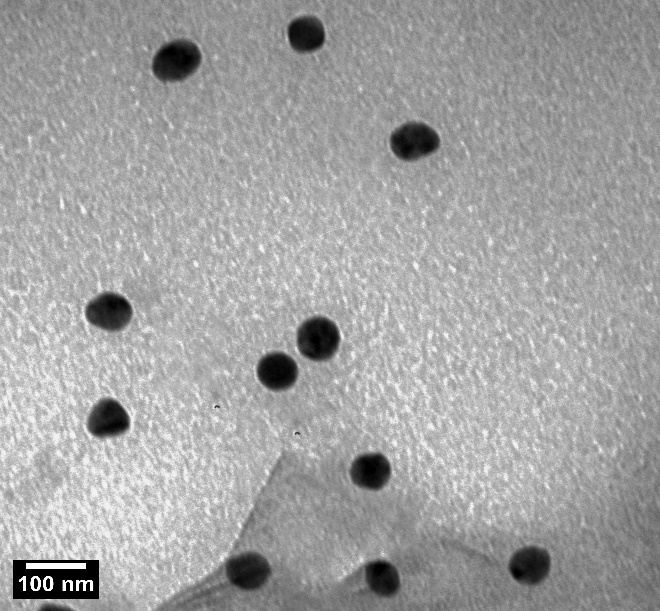


Supplementary Figure 2: HR-TEM of precipitated CaCO3 nanoparticles of isolate W39.

Supplementary Figure (3A) presents FE-SEM micrographs of the CaCO_3_ nanoparticles produced by the bacterial isolate, showing their spherical morphology along with pronounced surface agglomerations. The corresponding EDX spectra confirmed the elemental composition of the CaCO_3_ nanoparticles. The mass percentages of Ca, C, and O were 21.80%, 6.46%, and 61.42%, respectively, as illustrated in Supplementary Fig. (3B). This analysis confirms that the precipitated CaCO_3_ nanoparticles possess high purity.

These findings are consistent with previous reports by Frankel and Bazylinski [8], who demonstrated that mineral compositions resulting from biologically induced processes exhibit considerable heterogeneity, reflecting the diversity of formation environments. Furthermore, Gu et al. [9] described the morphological evolution of CaCO₃ during MICP, from early-stage spherical and irregular form to well-defined rhombohedral calcite crystal by 7 days. This transition reflects a phase shift from metastable vaterite to thermodynamically stable calcite. Such variability encompasses external morphology, often poorly defined, along with differences in water content, trace and minor element compositions, crystal structure, and particle size.


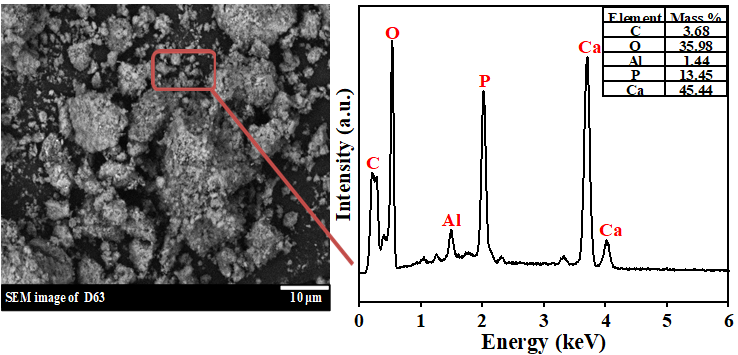


**B**

**A**

**Supplementary Figure 3:** The morphology of precipitated CaCO_3_ nanoparticles produced by isolate W39; FE-SEM images (A); EDX patterns of CaCO_3_ (B).

**3.5.3. Fourier-Transform Infrared Spectral Analysis**

Supplementary Figure 4 displays the FT-IR spectra of CaCO₃ precipitates formed by the bacterial isolate. The spectra exhibit three prominent absorption bands corresponding to the fundamental vibrational modes of the carbonate ion (CO₃²⁻), confirming the successful bio-mineralization of calcium carbonate. The peaks were observed at 1458.5 cm⁻¹, 1040.6 cm⁻¹, and 558.3 cm⁻¹; these absorption bands are attributed to the vibrational modes of the carbonate ion (CO₃²⁻). Specifically, the bands near 1450-1525 cm⁻¹ are typically associated with asymmetric stretching (ν₃) vibrations, while those around 1030-1040 cm⁻¹ correspond to symmetric stretching (ν₁), and the lower bands near 560-570 cm⁻¹ are linked to out-of-plane bending (ν₄) modes [10].


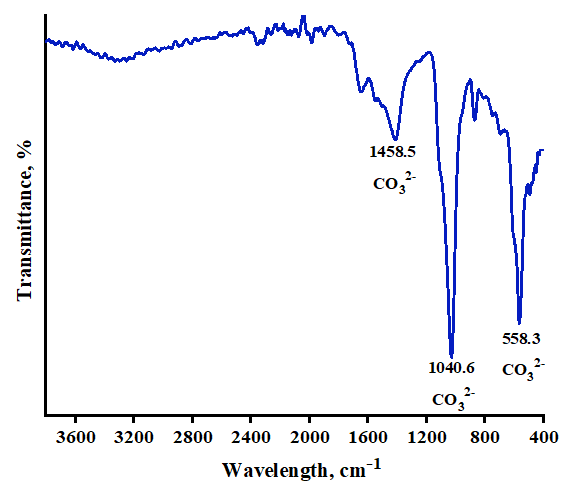


Supplementary Figure 4: FT-IR analysis of CaCO3 produced by isolate W39.

**3.5.4. Surface Area Characterization**

The N₂ adsorption/desorption isotherms and pore size distribution curves of the precipitated CaCO₃ are presented in Supplementary Figure 5. The N₂ adsorption/desorption isotherm exhibits a hysteresis loop at relative pressures (P/P^O^ ≈ 1), indicating the presence of extensive mesopores and macropores in the precipitates, which can be classified as type IV according to Brunauer’s categorization.

The BET surface area, average pore size, and pore volume of the CaCO₃ precipitate were determined to be 17.895 m²/g, 2.271 nm, and 0.1642 cc/g, respectively. The Barrett-Joyner-Halenda (BJH) pore size distribution, illustrated in Supplementary Fig. (5B), exhibits two distinct peaks: the first peak at 4.4 nm and the second peak at 6.7 nm. According to Qi et

al. [11]. These findings confirm that the precipitated CaCO₃ in both isolates predominantly comprises mesopores, following the classification criteria: micropores (0 nm<pore diameter<2 nm), mesopores (2nm<pore diameter<50 nm), and macropores (pore diameter> 50 nm).


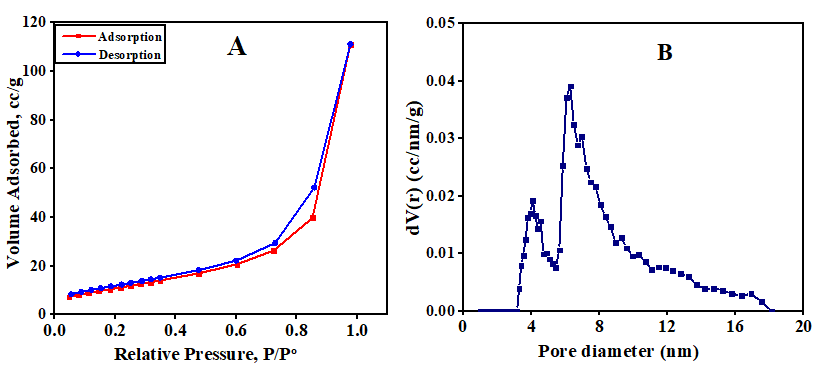


Supplementary Figure 5: N2 adsorption/desorption of CaCO3 (A), and (B) pore size distribution of CaCO3 produced by isolate W39.

**References**

1. Achal, V., Mukherjee, A., Basu, P. C. & Reddy, M. S. Lactose mother liquor as an alternative nutrient source for microbial concrete production by Sporosarcina pasteurii. *J. Ind. Microbiol. Biotechnol.* **36**, 433–438 (2009).

2. Achal, V., Mukherjee, A. & Reddy, M. S. Biocalcification by Sporosarcina pasteurii using corn steep liquor as the nutrient source. *Industrial Biotechnology* **6**, 170–174 (2010).

3. Abo-El-Enein, S. A. *et al.* Physico-mechanical properties of high performance concrete using different aggregates in presence of silica fume. *HBRC Journal* **10**, 43–48 (2014).

4. Wang, J. Y., Soens, H., Verstraete, W. & De Belie, N. Self-healing concrete by use of microencapsulated bacterial spores. *Cem. Concr. Res.* **56**, 139–152 (2014).

5. Fang, C., He, J., Achal, V. & Plaza, G. Tofu wastewater as efficient nutritional source in biocementation for improved mechanical strength of cement mortars. *Geomicrobiol. J.* **36**, 515–521 (2019).

6. Charpe, A. U., Latkar, M. V & Chakrabarti, T. Microbially assisted cementation--A biotechnological approach to improve mechanical properties of cement. *Constr. Build. Mater.* **135**, 472–476 (2017).

7. Sarkar, M., Chowdhury, T., Chattopadhyay, B., Gachhui, R. & Mandal, S. Autonomous bioremediation of a microbial protein (bioremediase) in Pozzolana cementitious composite. *J. Mater. Sci.* **49**, 4461–4468 (2014).

8. Frankel, R. B. & Bazylinski, D. A. Biologically induced mineralization by bacteria. *Rev. Mineral. Geochem.* **54**, 95–114 (2003).

9. Gu, Z. *et al.* Morphological changes of calcium carbonate and mechanical properties of samples during microbially induced carbonate precipitation (MICP). *Materials* **15**, 7754 (2022).

10. Ramasamy, V., Anand, P. & Suresh, G. Biomimetic synthesis and characterization of precipitated CaCO3 nanoparticles using different natural carbonate sources: A novel approach. *Int. J. Mater. Sci* **12**, 499–511 (2017).

11. Qi, S. *et al.* Investigation of Zn2+ and Cd2+ adsorption performance by different weathering basalts. *Water Air Soil Pollut.* **227**, 126 (2016).
